# Residential greenness attenuated association of long-term air pollution exposure with elevated blood pressure: Findings from polluted areas in Northern China

**DOI:** 10.3389/fpubh.2022.1019965

**Published:** 2022-09-29

**Authors:** Yayuan Mei, Jiaxin Zhao, Quan Zhou, Meiduo Zhao, Jing Xu, Yanbing Li, Kai Li, Qun Xu

**Affiliations:** ^1^Department of Epidemiology and Biostatistics, Institute of Basic Medical Sciences Chinese Academy of Medical Sciences, School of Basic Medicine Peking Union Medical College, Beijing, China; ^2^Center of Environmental and Health Sciences, Chinese Academy of Medical Sciences, Peking Union Medical College, Beijing, China

**Keywords:** air pollution, blood pressure, cross-sectional study, mixture, residential greenness

## Abstract

**Background:**

Evidence on the hypertensive effects of long-term air pollutants exposure are mixed, and the joint hypertensive effects of air pollutants are also unclear. Sparse evidence exists regarding the modifying role of residential greenness in such effects.

**Methods:**

A cross-sectional study was conducted in typically air-polluted areas in northern China. Particulate matter with diameter < 1 μm (PM_1_), particulate matter with diameter < 2.5 μm (PM_2.5_), particulate matter with diameter < 10 μm (PM_10_), nitrogen dioxide (NO_2_), sulfur dioxide (SO_2_), and ozone (O_3_) were predicted by space–time extremely randomized trees model. We used the Normalized Difference Vegetation Index (NDVI) to reflect residential green space. Systolic blood pressure (SBP) and diastolic blood pressure (DBP) were examined. We also calculated the pulse pressure (PP) and mean arterial pressure (MAP). Generalized additive model and quantile g-computation were, respectively, conducted to investigate individual and joint effects of air pollutants on blood pressure. Furthermore, beneficial effect of NDVI and its modification effect were explored.

**Results:**

Long-term air pollutants exposure was associated with elevated DBP and MAP. Specifically, we found a 10-μg/m^3^ increase in PM_2.5_, PM_10_, and SO_2_ were associated with 2.36% (95% CI: 0.97, 3.76), 1.51% (95% CI: 0.70, 2.34), and 3.54% (95% CI: 1.55, 5.56) increase in DBP; a 10-μg/m^3^ increase in PM_2.5_, PM_10_, and SO_2_ were associated with 1.84% (95% CI: 0.74, 2.96), 1.17% (95% CI: 0.52, 1.83), and 2.43% (95% CI: 0.71, 4.18) increase in MAP. Air pollutants mixture (one quantile increase) was positively associated with increased values of DBP (8.22%, 95% CI: 5.49, 11.02) and MAP (4.15%, 95% CI: 2.05, 6.30), respectively. These identified harmful effect of air pollutants mainly occurred among these lived with low NDVI values. And participants aged ≥50 years were more susceptible to the harmful effect of PM_2.5_ and PM_10_ compared to younger adults.

**Conclusions:**

Our study indicated the harmful effect of long-term exposure to air pollutants and these effects may be modified by living within higher green space place. These evidence suggest increasing residential greenness and air pollution control may have simultaneous effect on decreasing the risk of hypertension.

## Introduction

High blood pressure has been reported to play a vital role in a broad spectrum of renal and cardiovascular disease (CVD) and is one of the leading causes for the global burden of disease (1, 2). High systolic blood pressure is also regarded as the top risk factors for CVD, especially for the Chinese population (3). In recent years, the blood pressure levels has been reported to have increased in developing country population, which may related to the life style shift and the irritation of environmental toxic factors (4).

Concurrently, air pollution is pointed to be the largest global environmental threat to human health (5). Mechanistic evidence shows that air pollution exposure may induce the production of pro-inflammatory mediators and oxidative stress products, cause autonomic nervous imbalance, lead to abnormal DNA methylation status, and trigger endothelial dysfunction (6–10), which are also the underlying pathophysiological pathways of hypertension. Therefore, air pollution has been speculated to contribute to the development of high blood pressure. In the past decades, studies have explored the association between air pollutants exposure and blood pressure indicators (11–15).

However, current evidence regarding the hypertensive effect of air pollutants are mixed with some found positive associations (11, 13), some reported non-significant associations (16), and others showed reversed results (14). For example, Du et al., conducted a study among 23,256 participants aged 18–74 years found that an interquartile range increase in PM_2.5_ (16.1 μg/m^3^) was associated with 0.49 mmHg (95% CI: 0.22, 0.77) increase in diastolic blood pressure (DBP) (13). While another study reported an inversed association between PM_2.5_ exposure and DBP with effect estimate of −0.46 (95% CI: −0.68, −0.24) (14). Furthermore, previous study mainly focused on the effect of PM_2.5_, PM_10_, and NO_2_ on blood pressure (5). Evidence on the hypertensive effects of long-term exposure to PM_1_ and SO_2_ are scarce. Moreover, in the real world scenario, people are simultaneously exposed to various air pollutants, which puts a need to explore the joint effect of the general air pollutants. However, the joint hypertensive effects of air pollutants mixture are currently lacked. Finally, most previous studies were conducted in developed counties or general areas (5). Studies conducted in typically air polluted areas were scarce (17). For example, the Beijing-Tianjin-Hebei (BTH) area in China, where air pollution has been increasingly severe associated with related industrialization and urbanization (18). According to the “2019 Bulletin on the State of China's Ecological Environment” (19), the annual average of PM_2.5_, PM_10_, O_3_, SO_2_, and NO_2_ in 2019 were 57, 100, 196, 15, 40 μg/m^3^, respectively, all of which (except SO_2_) exceed the primary standard levels. Meanwhile, the BTH area has severe high hypertension prevalence. For example, the hypertension prevalence in Beijing (35.9%) ranked the top one province-level municipalities in China (20). Hebei also has a prevalence of hypertension with 23.3% higher than the nation level (23.2%) (20). Therefore, current research gap necessities a better understanding about the association between air pollutants exposure and blood pressure, particularly in typically air-polluted areas.

Previous studies have demonstrated the beneficial effect of residential greenness on human health such as birth outcomes (21), cardiovascular health (22), mental health (23) and so on. Recently, studies began to explore the potential benefits of residential greenness on hypertension risk and blood pressure (24, 25). However, existing evidence is limited. Moreover, few studies have explored the modifying role of residential greenness in the association between air pollutants and blood pressure. For example, study generally investigate the separate individual effect of air pollutants or greenness on blood pressure (16).

In this study, a cross-sectional design was adopted to investigate the individual and joint effect of long-term exposure to PM_1_, PM_2.5_, PM_10_, NO_2_, SO_2_, and O_3_ on blood pressure indicators in two Chinese cities with serious air pollution situations and high hypertension burden. Furthermore, we explored the potential beneficial effect of residential greenness and its modifying role in the air pollutants-blood pressure associations.

## Methods

### Study design and populations

We collected data from two typically air polluted cities (Beijing and Baoding) located in the BTH region from the northern China. Briefly, a multistage, stratified cluster sampling method was adopted among general population in these two cities between 2018 and 2020. First, to maximize the inter-district gradients of air pollutants, we selected one or two districts within each functional zone through simple cluster sampling, and seven districts were included for further sampling. Second, at least one community from each district was selected on the basis of its surrounding contamination conditions, population stability, and local medical conditions. Third, all permanent residents who provided signed informed consent in these communities were selected as the study sample.

Inclusion criteria were those aged ≥ 18 years and living in their community for more than 5 years. Ineligible participants were those with cancer, major cardiovascular diseases, mental disorders, and pregnant women. Meanwhile, participants who could not provide necessary information were excluded. On enrollment, participants completed the survey by a team of professionals. Trained investigators collected information regarding demographic and lifestyle factors and disease status. Qualified physicians were responsible for collecting anthropometric measurements, and certified nurses collected blood samples. All the procedures were in accordance with the standard operating procedures. Participants provided written informed consent prior to study enrollment, and the Institutional Review Board of the Institute of Basic Medical Sciences, Chinese Academy of Medical Sciences approved this study protocol (No. 029-2015).

### Air pollutants assessment

Grid data on particulate matters (PM_1_, PM_2.5_, PM_10_) with the spatial resolution of 1 × 1 km, and gaseous pollutants (NO_2_, SO_2_, and O_3_) with the spatial resolution of 10 × 10 km in our study sites were obtained from the validated ChinaHighAirPollutants (CHAP) dataset, which was developed using machine learning prediction model (26–29). These data sets exhibited high predictive ability for daily measurements, with 10-fold cross-validation root-mean-square error (*R*^2^) values of 14.6 μg/m^3^ (0.77) for PM_1_, 5.07 μg/m^3^ (0.94) for PM_2.5_, 24.28 μg/m^3^ (0.86) for PM_10_, 7.99 μg/m^3^ (0.84) for NO_2_, 10.07 μg/m^3^ (0.84) for SO_2_, and 17.10 μg/m^3^ (0.87) for O_3_. The 1-year average levels of PM_1_, PM_2.5_, PM_10_, NO_2_, SO_2_, and O_3_ before the baseline survey year for each participant were calculated on the basis of the geocode of their address to represent their long-term air pollutants exposure.

### Residential greenness

We used the Normalized Difference Vegetation Index (NDVI) to reflect the residential surrounding greenness for each participant. NDVI has been widely adopted to investigate the health effects of greenness, with values ranged from −0.2 to +1.0 (30–32). The higher values represented greater vegetation greenness. NDVI is estimated using the land surface reflectance of the visible red (500–650 nm) and near infrared band (700–900 nm). In this study, we adopted the max value of NDVI during a year to indicate the long-term residential greenness exposure, which was obtained from the National Ecosystem Science Data Center, National Science & Technology Infrastructure of China (http://www.nesdc.org.cn) (33). 0.25 miles (about 400 m) has been proposed to be a suitable radius distance to estimate accessible greenness (31). Meanwhile, studies have widely adopted 500 m radius of greenness to explore the effects of greenness on human health (30–32). Therefore, we calculated the mean values of the max NDVI in 500 m circular buffer for participants. We assigned each participants' residential greenness exposure according to their residential location.

### Outcome assessment and definition

We measured the blood pressure for each participant according to qualified SOPs. Participants were prohibited from exercising and were told not to consume tea, coffee, tobacco, and alcohol for 30 min before the examination. Briefly, blood pressure was measured with a calibrated mercury sphygmomanometer after a 15-min rest period by qualified physicians. A minimum of three measurements at intervals of >2 min were recorded until the difference between successive measurements was < 5 mmHg. The mean values from the last two readings were calculated to determine systolic BP (SBP) and diastolic BP (DBP). Furthermore, we also calculated the pulse pressure (PP) and mean arterial pressure (MAP) using the following equation (34): *PP* = *SBP*−*DBP*; *MAP* = [(2 × *DBP*)+*SBP*]/3.

### Covariates

Standard questionnaires were used by trained investigators to collect the following covariates data information. Age and sex were extracted from their identification card. Age was treated as a continuous variable. Sex was regarded as a category variable (male/female). Educational attainment was classified as primary school or less, junior or senior high school, or college or higher. For cigarette use, participants were categorized into non-smokers, current smoker, and former smoker. Current smokers were defined as subjects who smoked at least one cigarette per day over the last 6 months; while participants who had ceased smoking more than 6 months prior were considered former smokers. For alcohol consumption, participants were divided into non-drinker, current drinker (who consumed alcohol at least once per week over the previous 6 months), and former drinker (who had ceased drinking more than 6 months prior). Height and weight were measured with participants wearing light clothing and no shoes. BMI was calculated as weight (kg) divided by height (m) squared. Hypertension was defined as SBP ≥140 mmHg or DBP ≥90 mmHg or using antihypertensive agents (35), or have been previously diagnosed by doctor. Diabetes was defined as fasting blood glucose ≥7 mmol/L, or physician diagnosed history, or intake of any antidiabetic agents (36, 37). Dyslipidemia was defined as total cholesterol ≥6.22 mmol/L, triglyceride ≥2.26 mmol/L, high-density lipoprotein cholesterol ≤ 1.04 mmol/L, low-density lipoprotein cholesterol ≥4.1 mmol/L (38), or the use of any antihyperlipidemic agent. Due to the health effect of temperature and relative humidity (39), we also collected daily mean temperature and relative humidity data from the China Meteorological Data Network (http://data.cma.cn/).

### Statistical analysis

**Figure 1:**
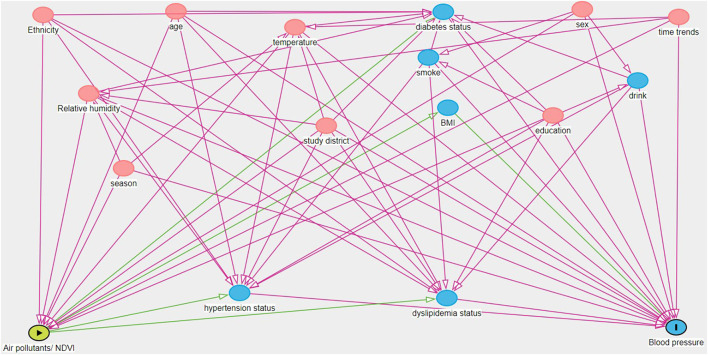
Directed acyclic graph (DAG) showing the relationship between exposure variable (the green oval with the triangle) and blood pressure (the blue oval with the line). Covariates with pink ovals are ancestors of exposure and outcome, while variables with blue ovals are ancestors only for the outcome. The minimum adjustment set include age, sex, ethnicity, education, temperature, relative humidity, season, study district, and time trends (For interpretation of the references to color in this figure legend, the reader is referred to the web version of this article).

Mean ± standard deviations (SD) and number (percentage) were presented to describe the continuous and categorical variables, respectively. Pair wise correlations between exposure variables were examined by the spearman's rank correlation test. We employed the generalized additive model to investigate the associations of air pollutants and NDVI with blood pressure indicators, which were log–transformed to increase their conformity to normal distributions of residuals. Percent changes in the outcomes were calculated with per 10 μg/m^3^ and 0.1 unit increase in air pollutants and NDVI, respectively. The equation is [exp(β × *per increasement*)−1] × 100%, where the β is the effect estimate. We employed a progressive cofounder adjustment and thus constructed three models. Model 1: we adjusted the minimal adjustment covariates set identified by the direct acyclic graph (DAG) in case of multicollinearity and overadjustment (Figure 1). However, study proposed that covariates that can only influence the exposure or outcome, which cannot identified by DAG, should also be included in the model (40). For example, the BMI, smoking habit, drinking habit, diabetes history, hypertension history, and dyslipidemia history in our study. Moreover, some covariates such as BMI and disease history may act either as confounder, mediator, or collider. Therefore, we further constructed the Model 2 that added smoking and drinking habits based on Model 1. Model 3 (also the core model) was based on Model 2 and further adjusted BMI and disease history. Therefore, we controlled the following covariates in the core model, including age, sex, BMI, ethnicity, educational level, temperature, relative humidity, season, study district, month of blood sample collection, smoking status, drinking habits, and disease history of hypertension, dyslipidemia and diabetes. Air pollutant levels were separately incorporated as a linear term. Age, BMI, temperature, and relative humidity were controlled by smooth terms to account for their potentially nonlinear effects. The month of blood sample collection was included with a smooth term to control for nonmonotonic changes and secular trends in outcomes (41). The study districts were included with a random-effect term. In this study, we mainly focused on the results from the core model.

Quantile g-computation was adopted to explore the joint effect of air pollutants on each blood pressure indicator. The covariates in the quantile g-computation model were the same as the individual effect analysis. This kind of model can get effect estimate with one-quantile increases in the air pollutants mixture. It does not require all exposure variables in the mixture have the same effect direction with the outcome, and thus can capture the nonlinear, nonadditive effects of both individual and mixture of environmental pollutants (42). Meanwhile, this method has been widely used to explore the effects of air pollutants mixture on human health (42, 43). Furthermore, to evaluate the protective effect of residential greenness, we added the NDVI to the mixture model.

We performed stratified analysis by age (< 50 or ≥ 50 years), sex (male or female), smoking status (yes or no), and drinking status (yes or no). The differences between strata were significant by calculating 95% CIs as follows:


$$\begin{array}{l} (\beta_{1}-\beta_{2})\pm 1.96\times \sqrt{SE_{1}{\;}^{2}\;+\;SE_{2}{\;}^{2}\;} \end{array}$$

where β_1_ and β_2_ are the effect estimates of each stratification and SE_1_ and SE_2_ are their corresponding standard errors, respectively (37). Furthermore, to test the potential modification effect of residential greenness, we further classified NDVI in to low NDVI group (smaller than median value) and high NDVI group (larger than median value) and further explore the effect of air pollutants on blood pressure. Then, a product interaction term between air pollutants and dichotomous terms for low and high NDVI were included in the model. *P*-values for the product terms were used to test differences association between each group (44).

Several sensitivity analyses were performed to evaluate the robustness of our results, including: (1) using 2-years and 5-years average concentration before the year of health examination as the long-term exposure metrics. Due to the data restriction of PM_1_, we adopted the 4-year average levels for PM_1_ in the 5-year sensitivity analysis; (2) excluding outliers defined as values out of four standard deviations (SDs) from the mean; (3) restricting analysis to participants without hypertension, diabetes, and dyslipidemia. All analyses were performed using R version 4.1.1 (R Foundation for Statistical Computing, Vienna Austria). A two sided *P*-value of < 0.05 indicate statistical significance.

## Results

Table 1 shows the demographic characteristics of the study participants. In this study, 4,235 participants were included in our analysis. These participants have a mean (SD) age of 54.23 (14.66) years, and the number of men (49.68%) were generally comparable with the number of women (50.32%). About 45.88% of the participants had junior or senior high school educational level. The majority of participants were non-smoker (66.97%) and non-drinker (53.70%). The mean (SD) value of the blood pressure indicators were 134.41 (19.40) mmHg for SBP, 79.48 (11.19) mmHg for DBP, 97.79 (12.59) mmHg for MAP, 54.93 (15.07) mmHg for PP, respectively.

**Table 1:** Characteristic information of the participants.

**Variables**	**Mean ±SD or *n* (%)**
**Demographic characteristics**	
No.	4,235
Age, years	54.23 ± 14.66
BMI, kg/m^2^	25.30 ± 3.05
**Sex**	
Male	2,104 (49.68)
Female	2,131 (50.32)
**Ethnicity**	
Han	4,086 (96.48)
other	149 (3.52)
**Education**	
Primary school or below	998 (23.57)
Junior or senior high school	1,943 (45.88)
College or higher	1,294 (30.55)
**Smoke**	
Never	2,836 (66.97)
Current	1,063 (25.10)
Former	336 (7.93)
**Drink**	
Never	2,274 (53.70)
Current	1,780 (42.03)
Former	181 (4.27)
**Hypertension**	
Yes	2,074 (48.97)
No	2,161 (51.03)
**Diabetes**	
Yes	599 (14.14)
No	3,636 (85.86)
**Dyslipidemia**	
Yes	1,608 (37.97)
No	2,627 (62.03)
**Meteorological factors**	
Relative humidity (%)	64.77 ± 14.73
Temperature (°C)	20.56 ± 7.50
**Blood pressure indicators**	
SBP (mmHg)	134.41 ± 19.40
DBP (mmHg)	79.48 ± 11.19
MAP (mmHg)	97.79 ± 12.59
PP (mmHg)	54.93 ± 15.07

BMI, body mass index; SD, standard deviation. SBP, systolic blood pressure; DBP, diastolic blood pressure; MAP, mean arterial pressure; PP, pulse pressure.

Table 2 presents the distribution of the long-term exposure to each air pollutant and residential greenness. The average (SD) levels of the 1-year exposure were 32.98 (9.51) μg/m^3^ for PM_1_, 51.77 (20.56) μg/m^3^ for PM_2.5_, 98.05 (28.02) μg/m^3^ for PM_10_, 39.08 (7.66) μg/m^3^ for NO_2_, 17.15 (7.31) μg/m^3^ for SO_2_, and 104.44 (5.50) μg/m^3^ for O_3_. The mean levels of particulate matters such as PM_2.5_ and PM_10_ were much higher than the primary standard of annual average for China. O_3_ concentrations were also much higher than the latest WHO guidelines. While, the 1-year average concentration of SO_2_ and NO_2_ did not exceed the Chinese standards.

**Table 2:** Summary of the residential greenness and 1-year air pollutants exposure of the participants.

**Exposure variables**	**Mean (SD)**	**Median (IQR)**	China standard^*^	**% of > China standard**	**WHO guideline^#^**	**% of > WHO guideline**
**Air pollutants**						
PM_1_, μg/m^3^	32.98 ± 9.51	31.58 (23.14, 43.77)	None	None	None	None
PM_2.5_, μg/m^3^	51.77 ± 20.56	47.25 (31.64, 78.03)	15.00	100.00	5.00	100.00
PM_10_, μg/m^3^	98.05 ± 28.02	99.24 (73.77, 133.37)	40.00	100.00	15.00	100.00
NO_2_, μg/m^3^	39.08 ± 7.66	39.76 (31.61, 48.32)	40.00	49.99	10.00	100.00
SO_2_, μg/m^3^	17.15 ± 7.31	12.99 (11.49, 26.79)	20.00	36.32	None	None
O_3_, μg/m^3^	104.44 ± 5.50	103.25 (99.90, 111.12)	None	None	60.00	100.00
**Residential greenness**						
NDVI	0.42 ± 0.11	0.41 (0.35, 0.48)	None	None	None	None

SD, standard deviation; Min, minimum; Max, maximum. PM_1_, particulate matter with a diameter of < 1 μm; PM_2.5_, fine particulate matter of < 2.5 μm; PM_10_, particulate matter with a diameter of < 10 μm; NO_2_, nitrogen dioxide; SO_2_, sulfur dioxide; O_3_, ozone; SBP, systolic blood pressure; DBP, diastolic blood pressure; MAP, mean arterial pressure; PP, pulse pressure. NDVI, Normalized Difference Vegetation Index.

* Primary standard levels for annual average proposed by China's Ministry of Ecology and Environment.

# Recommended air quality guideline for long-term standard levels (annual average for PM_2.5_, PM_10_, NO_2_; peak season for O_3_) by world health organization.

Supplementary Figure S1 shows the spearman's correlation analysis. Pollutants were strongly correlated with each other correlation coefficients ranging from 0.85 to 0.99. While NDVI was weakly to moderately correlated with air pollutants with correlation coefficients ranging from 0.25 to 0.57.

**Figure 2:**
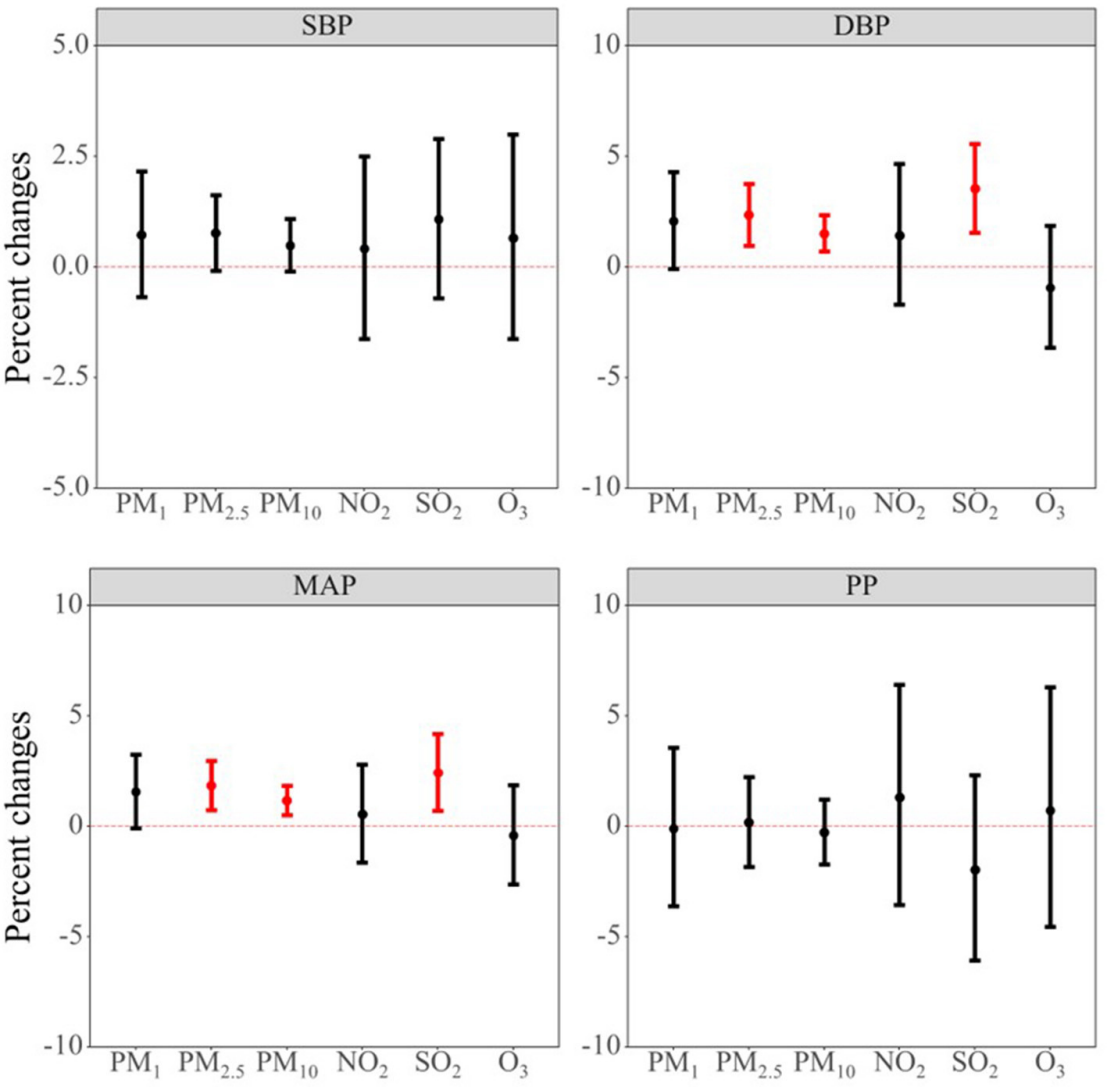
Associations between per 10-μg/m^3^ increment in air pollutants and blood pressure indicators. Abbreviations: PM1, particulate matter with a diameter of < 1 μm; PM_2.5_, fine particulate matter of < 2.5 μm; PM_10_, particulate matter with a diameter of < 10 μm; NO_2_, nitrogen dioxide; SO_2_, sulphur dioxide; O_3_, ozone; SBP, systolic blood pressure; DBP, diastolic blood pressure; MAP, mean arterial pressure; PP, pulse pressure.

Figure 2 shows the association between a 10-μg/m^3^ increase in long-term air pollutants exposure and blood pressure indicators. We found that long-term air pollutants exposure was mainly associated with elevated values of DBP and MAP. To be more specific, we found that a 10-μg/m^3^ increase in PM_2.5_, PM_10_, and SO_2_ were associated with 2.36% (95% CI: 0.97, 3.76), 1.51% (95% CI: 0.70, 2.34), and 3.54% (95% CI: 1.55, 5.56) increase in DBP. A 10-μg/m^3^ increase in PM_2.5_, PM_10_, and SO_2_ were also found to be associated with 1.84% (95% CI: 0.74, 2.96), 1.17% (95% CI: 0.52, 1.83), and 2.43% (95% CI: 0.71, 4.18) increase in MAP. Results from the Model 1 and Model 2 were generally consistent with the results from the core model with similar effect estimate and direction (see Supplementary Table S1 for details).

Supplementary Figure S2 presents the results of the association between 0.1 unit increase in NDVI and blood pressure indicators. No significant associations were found between NDVI and blood pressure indicators. This is consistent with the results from the Model 1 and Model 2, which is presented in Supplementary Table S2.

**Figure 3:**
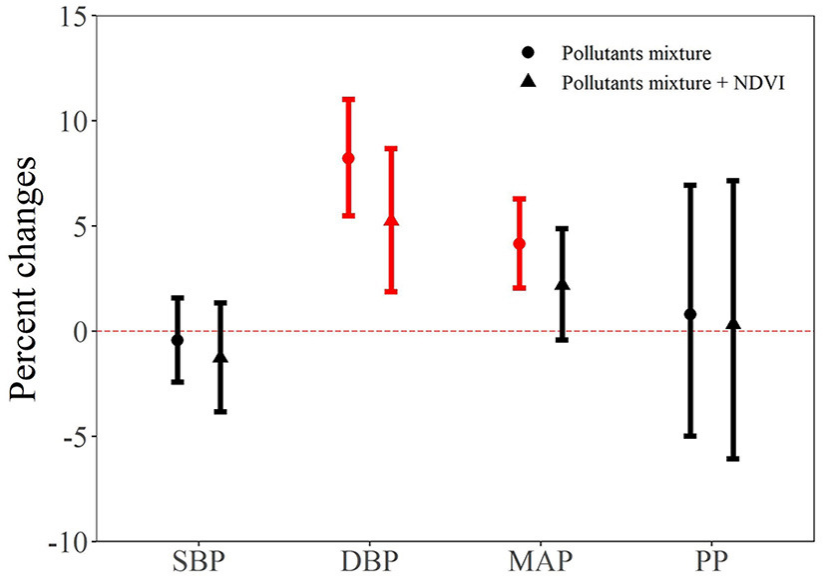
The joint effect of air pollutants on blood pressure indicators. NDVI, Normalized Difference Vegetation Index; SBP, systolic blood pressure; DBP, diastolic blood pressure; MAP, mean arterial pressure; PP, pulse pressure.

Figure 3 shows the joint effects of air pollutants mixture on each blood pressure indicator. We found that air pollutants mixture was positively associated with increased values of DBP and MAP. Specifically, one quantile increase in the six air pollutants mixture was associated with 8.22% (95% CI: 5.49, 11.02) and 4.15% (95% CI: 2.05, 6.30) increase in DBP and MAP, respectively. Furthermore, we added the NDVI in the mixture model to test the potential protective effect. As shown in Figure 3, we can see that the effect estimate of air pollutants mixture on each blood pressure indicator were all became smaller. For example, the effect of air pollutants mixture on MAP became nonsignificant (2.19%, 95% CI: −0.42, 4.88) after adding the NDVI in the model.

We also did stratified analysis by age, sex, smoking habits, and drinking habits (see Tables 3, 4; Supplementary Tables S3–S7 for details). We can see that the stratified results were consistent with the results form the core model. Take the effect of PM_2.5_ and SO_2_ for example, we can notice that significant results mainly appeared in DBP and MAP. After the subgroup significant test, we found participants aged ≥50 years are more susceptible to the harmful effect of PM_2.5_ and PM_10_ compared to younger adults. For example, 10-μg/m^3^ increase in PM_2.5_ was associated with 0.61% (95% CI: −0.25, 1.47) increase among subjects aged < 50 years and 3.09% (95% CI: 1.47, 4.73) increase among participants aged ≥50 years in DBP, respectively. Similarly, 10-μg/m^3^ increase in PM_2.5_ was associated with 0.51% (95% CI: 0.01, 1.00) increase among subjects aged < 50 years and 2.37% (95% CI: 1.07, 3.68) increase among participants aged ≥50 years in MAP, respectively.

**Table 3:** Stratified analysis of the association between 10-μg/m^3^ increase in PM_2.5_ and blood pressure indicators by potential modifiers.

**Stratification factors**	**Percent changes (95% CI)**			
	**SBP**	**DBP**	**MAP**	**PP**
**Age**				
< 50	0.43 (−0.09, 0.95)	0.61 (−0.25, 1.47)	**0.51 (0.01, 1.00)**	0.97 (−0.14, 2.08)
≥50	**1.11 (0.00, 2.22)**	3.09 (1.47, 4.73)*	2.37 (1.07, 3.68)*	−0.23 (−2.55, 2.15)
**Sex**				
Male	0.44 (−0.53, 1.41)	**1.76 (0.15, 3.40)**	1.06 (−0.04, 2.17)	−0.30 (−2.84, 2.31)
Female	0.41 (−0.55, 1.38)	**1.58 (0.08, 3.11)**	**1.37 (0.17, 2.58)**	0.41 (−1.64, 2.50)
**Smoking**				
No	0.58 (−0.37, 1.55)	**2.06 (0.61, 3.54)**	**1.83 (0.65, 3.03)**	0.23 (−1.61, 2.10)
Yes	0.52 (−0.30, 1.35)	1.19 (−0.44, 2.85)	0.75 (−0.23, 1.74)	0.17 (−2.18, 2.57)
**Drinking**				
No	0.57 (−0.34, 1.48)	**1.51 (0.06, 2.98)**	**1.25 (0.15, 2.36)**	0.67 (−1.22, 2.60)
Yes	0.59 (−0.51, 1.69)	1.28 (−0.15, 2.73)	1.06 (−0.13, 2.27)	−0.13 (−2.95, 2.76)

PM_2.5_, fine particulate matter of < 2.5 μm; SBP, systolic blood pressure; DBP, diastolic blood pressure; MAP, mean arterial pressure; PP, pulse pressure. The asterisk indicate there is significant difference between the subgroups; no asterisk indicate there is no significant difference between the subgroups. Bold values indicate the effect estimates were significant.

**Table 4:** Stratified analysis of the association between 10-μg/m^3^ increase in SO_2_ and blood pressure indicators by potential modifiers.

**Stratification factors**	**Percent changes (95% CI)**			
	**SBP**	**DBP**	**MAP**	**PP**
**Age**				
< 50	1.36 (−0.31, 3.05)	**2.08 (0.19, 4.00)**	**1.83 (0.21, 3.48)**	2.09 (−1.39, 5.70)
≥50	1.72 (−0.5, 4.00)	**5.06 (2.54, 7.64)**	**3.64 (1.45, 5.88)**	−2.74 (−7.44, 2.19)
**Sex**				
Male	0.57 (−1.61, 2.80)	**3.18 (0.90, 5.51)**	**2.11 (0.19, 4.07)**	−3.98 (−9.15, 1.49)
Female	0.92 (−1.26, 3.16)	**3.23 (0.73, 5.80)**	**2.44 (0.27, 4.66)**	−0.41 (−5.09, 4.50)
**Smoking**				
No	0.97 (−1.08, 3.05)	**3.44 (1.27, 5.66)**	**2.48 (0.53, 4.46)**	−1.06 (−5.2, 3.26)
Yes	0.95 (−1.15, 3.10)	**3.35 (0.42, 6.37)**	2.11 (−0.04, 4.30)	−3.83 (−9.78, 2.50)
**Drinking**				
No	1.34 (−0.70, 3.44)	**3.35 (0.95, 5.80)**	**2.53 (0.54, 4.55)**	−0.54 (−5.08, 4.22)
Yes	0.27 (−2.24, 2.83)	**2.57 (0.14, 5.07)**	1.68 (−0.49, 3.90)	−3.71 (−9.37, 2.31)

SO_2_, sulfur dioxide; SBP, systolic blood pressure; DBP, diastolic blood pressure; MAP, mean arterial pressure; PP, pulse pressure. The asterisk indicate there is significant difference between the subgroups; no asterisk indicate there is no significant difference between the subgroups. Bold values indicate the effect estimates were significant.

**Figure 4:**
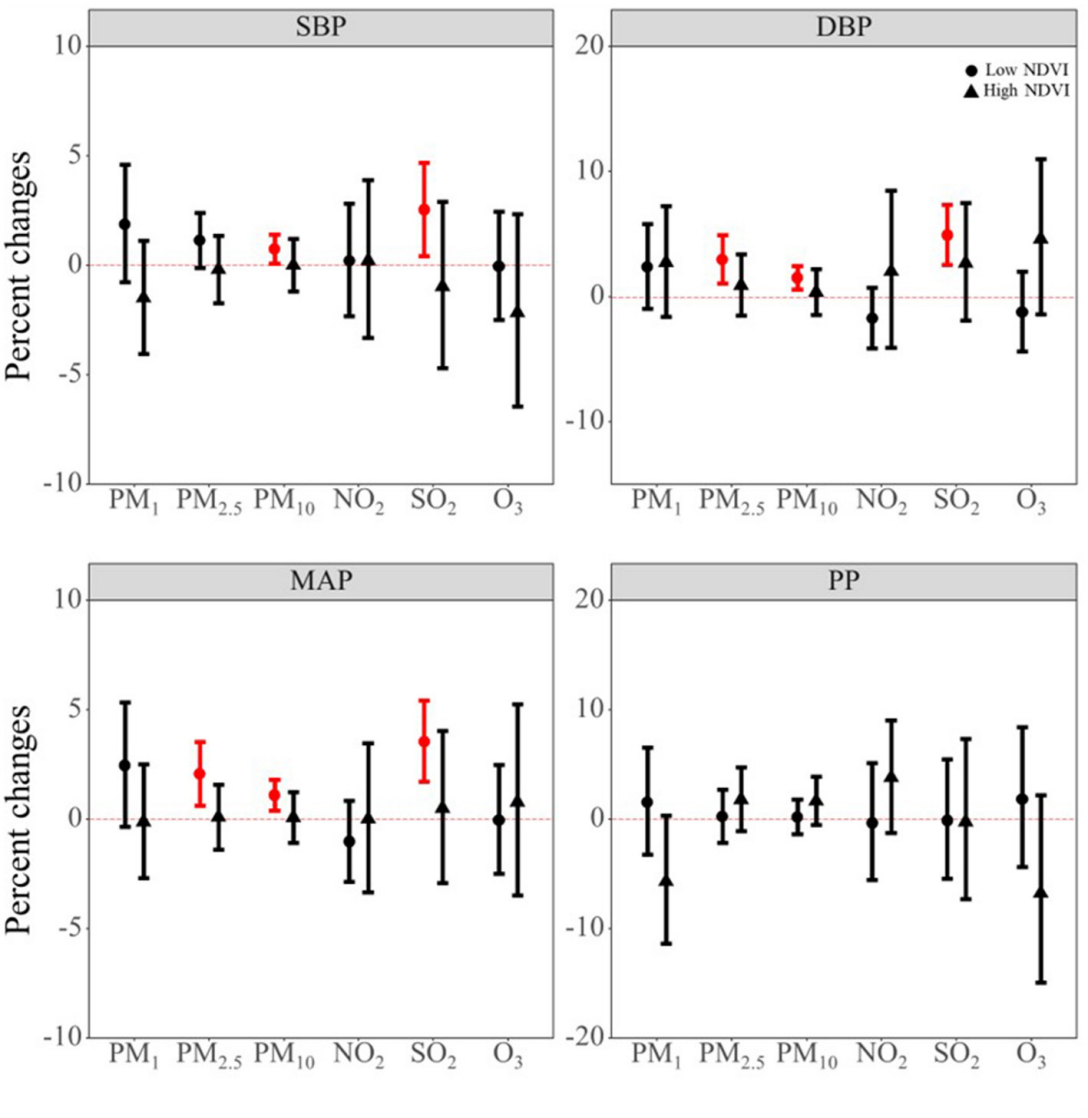
The potential modifying effect of NDVI on the association between air pollutants and blood pressure indicators. PM_1_, particulate matter with a diameter of < 1 μm; PM_2.5_, fine particulate matter of < 2.5 μm; PM_10_, particulate matter with a diameter of < 10 μm; NO_2_, nitrogen dioxide; SO_2_, sulphur dioxide; O_3_, ozone; NDVI, Normalized Difference Vegetation Index; SBP, systolic blood pressure; DBP, diastolic blood pressure; MAP, mean arterial pressure; PP, pulse pressure.

Although we did not find the individual significant protective effect of NDVI on blood pressure indicators, we observed a potential protective role of NDVI in the mixture model. Therefore, NDVI may be an important effect modifier for the air pollutant effect. Thus, we did stratified analysis by dividing the study population into NDVI high exposure group and NDVI low exposure group according to its median value. As shown in Figure 4, we can see that the effect of air pollutants on blood pressure main occurred in low NDVI exposure group. Supplementary Table S8 shows the *P*-values for the interaction term between air pollutants and dichotomous terms for NDVI. The results show that NDVI may interact with air pollutants. Combined with the results of stratified by low or high exposure of NDVI, we found that NDVI may have an interaction effect with PM_2.5_ and SO_2_ on the DBP, and with SO_2_ on MAP.

Supplementary Figures S3–S6 shows results for the sensitive analysis. We can notice that our results generally remained robust in these sensitive analysis. For example, when we used the 2- and 5-year exposure windows, we observed long-term air pollutants exposure was mainly associated with elevated values of DBP and MAP, which is consistent with the results form the core model. We also found positive associations between long-term PM_1_ exposure and increased levels of DBP and MAP in the 5-year sensitive analysis.

## Discussion

### Mian findings

In this study, we explored the individual and joint effect of air pollutants on blood pressure indicators. We observed positive associations of PM_2.5_, PM_10_, and SO_2_ with DBP and MAP. The joint effect of air pollutants mixture were also on DBP and MAP. Meanwhile, participants aged ≥50 years were more susceptible to the harmful effect of PM_2.5_ and PM_10_ compared to younger adults. Finally, NDVI modified these associations, thus suggesting a protective effect of residential greenness on the harmful air pollutants effects.

### Compared with previous studies

SBP has been a better predictor for risk compared to DBP, and has become the most common form for hypertension given its nature of increasing with age and the aging societies (45). In our study, we found no significant associations between any air pollutants and SBP. Previous studies regarding the effect of long-term air pollutants exposure on SBP were mixed. For example, a cross-sectional study with 23,256 participants aged 18–74 years from communities in China found that an interquartile range increase in PM_2.5_ (16.1 μg/m^3^), PM_10_ (19.3 μg/m^3^), NO_2_ (19.8 μg/m^3^), and SO_2_ (3.9 μg/m^3^) with associated with 1.86 mmHg (95% CI: 1.50, 2.22), 0.64 mmHg (95% CI: 0.26, 1.03), 0.57 mmHg (95% CI: 0.10, 1.05), and 1.74 mmHg (95% CI: 1.40, 2.09) changes in SBP, respectively (13). Another study conducted among 24,845 participants aged 18–74 years from 33 communities in China reported that a 10-μg/m^3^ increase in PM_1_ was associated with 0.57 mmHg (95% CI: 0.31, 0.83) increase in SBP (46). However, a study conducted among 1,432 participants aged 12 years reported no significant association between long-term air pollutants exposure and SBP (47). Similarly, a cross-sectional study in 27,752 residents aged > 65 years showed that none of the long-term air pollutants (PM_2.5_, PM_10_, PM_2.5 − 10_, NO_x_, NO_2_) exposure were associated with SBP (48). The seemingly contrary results from these studies may come from the heterogeneity in study design, study population characteristics, long-term exposure window definition and so on. Nevertheless, results from a comprehensive meta-analysis regarding the global association between air pollution and blood pressure were consistent with ours. This meta-analysis indicated non-significant associations of long-term air pollutants (PM_2.5_, PM_10_, PM_2.5 − 10_, NO_x_, NO_2_, O_3_) exposure with SBP (5).

DBP has traditionally been regarded as the most vital component of blood pressure and the primary aim of antihypertensive therapy (45). In our study, we found that a 10-μg/m^3^ increase in long-term PM_2.5_ and PM_10_ exposure were associated with 2.36% (95% CI: 0.97, 3.76) and 1.51% (95% CI: 0.70, 2.34) increase in DBP, respectively. Previous evidence were generally consistent with our findings. For example, a study conducted among 12,665 participants aged 50 years and older found that each 10 μg/m^3^ increase in PM_2.5_ was associated 1.04 mmHg (95% CI: 0.31, 1.78) changes in DBP (49). Another study also found such positive associations (50). Also, a study among 24,845 adults in 11 districts in China found that an interquartile range increase in long-term PM_10_ (19 μg/m^3^) exposure was associated 0.32 mmHg increase in DBP (51). All in all, current evidence showed a robust association of long-term exposure to PM_2.5_ and PM_10_ with increased levels of DBP. A comprehensive meta-analysis showed that 10 μg/m^3^ increase in PM_2.5_ and PM_10_ were associated with 0.47 mmHg (95% CI: 0.12, 0.82) and 0.86 mmHg (95% CI: 0.37, 1.35) increase in DBP (5). In our study, we also found that a 10-μg/m^3^ increase in long-term SO_2_ exposure was associated with 3.54% (95% CI: 1.55, 5.56) increase in DBP. It is interested that the observed significant results was under the situation that the mean exposure levels of SO_2_ (17.15 μg/m^3^) were below the Chinese primary standard levels (20 μg/m^3^). It suggested a more stringent standards for SO_2_ in China. Previous studies seldom explored the long-term SO_2_ exposure and blood pressure. However, existing evidence are consistent with our findings. For example, a study conducted in 11 Chinese districts involving 24,845 adults reported that each 20 μg/m^3^ increase in SO_2_ was associated with 0.31 mmHg (95% CI: 0.10, 0.51) increase in DBP. Previous evidence also showed that short-term SO_2_ exposure with positively associated with DBP (5).

MAP is calculated using the SBP and DBP, and it can simultaneously reflect peripheral vascular resistance and cardiac output during a cardiac cycle (52). Meanwhile, this indicator was associated with major cardiovascular events (53, 54). Similar to the results of DBP, we found that a 10-μg/m^3^ increase in PM_2.5_, PM_10_, and SO_2_ were associated with 1.84% (95% CI: 0.74, 2.96), 1.17% (95% CI: 0.52, 1.83), and 2.43% (95% CI: 0.71, 4.18) increase in MAP, respectively. Some of the previous studies are consistent with our findings (11, 55), while some are not (56, 57). For example, Chan et al., analyzed the Sister study data and found significant association of PM_2.5_ with MAP and nonsignificant association of NO_2_ with MAP, which supported our findings (11). While Honda et al., conducted a study among older Americans aged ≥57 years did not find significant association between 1-year moving concentrations of PM_2.5_ and MAP (0.32 mmHg, 95%CI: −0.24, 0.88) (56). The heterogeneous results may be due to the air pollutants concentration difference and population characteristic difference. In our study, the mean value of PM_2.5_ were 51.77 μg/m^3^, which is much higher than that of Honda et al., Moreover, the mean age of our study are about 54 years, while the study of Honda et al., has the mean age of about 70 years.

PP can reflect the stiffening of large arteries and is correlated with some cardiovascular risk factors (45). In our study, we did not find any significant associations between air pollutants and PP. Similar to our results, a previous longitudinal study also find non-significant associations of long-term PM_2.5_ and O_3_ exposure with increased PP values (14). This is reasonable, because previous study has indicated that although PP can predict cardiovascular events in epidemiological studies, its independent role is hampered by the close correlation between PP and SBP (45). And in our study we also did not find significant results for SBP. Another point should be noted that although previous studies have reported harmful health effect of O_3_ (58, 59), we did not found significant association between long-term O_3_ exposure and blood pressure in the main analysis. However, we found negative associations between O_3_ exposure and DBP and MAP in the 5-year analysis. Previous epidemiological study also found short-term O_3_ exposure was associated with decreased levels of blood pressure indicators (60). Similarly, experimental study indicated that O_3_ treatment may decrease blood pressure and prevent hypertension progression with the mechanisms of reducing the levels of serum endothelin-1 and ET receptor A mRNA expression (61).

### Potential mechanisms

Several potential mechanisms about the air pollutants exposure contributing to the hypertension development or increased blood pressure indicators have been proposed. First, autonomic nervous system may be triggered after air pollutants exposure, and then favor sympathetic over parasympathetic tone, and finally increase blood pressure (9). Second, air pollutants may also induce the creation and circulation of endogenous pro-inflammatory markers and vasculo-active molecules such as endothelin (7, 10) to influence vascular endothelium and then elevate blood pressure levels. Particulate matter contains various constituents including black carbon, metals and so on, which can be inhaled by human. Study has shown that the internal metal exposure were associated with the inflammatory homeostasis disorder (62). Third, air pollution may lead to abnormal DNA methylation status, which has been an explored mechanisms for the effect of air pollutants on blood pressure (6, 63).

Several mechanisms have also been proposed for the underlying effects of residential greenness on blood pressure. Greenness can remove air pollutants (64), which has been reported to be associated with elevated blood pressure both in previous studies and present study (11, 51). Furthermore, greenness can also encourage exercise and further influence participants' obesity status (65), which are strong risk factor for high blood pressure. Lastly, residential greenness may also alleviate personal stress, promote social cohesion, reduce surrounding noise and heat effects, and enrich microbial (66, 67).

### Strengths and limitations

Two key strengths of this study were the comprehensive exploration about the individual and joint effect of air pollutants, and the protective effect of residential greenness. Additionally, a series of sensitive analysis indicated that the results of our study are robust.

However, our findings should be interpreted with caution in light of the limitations. First, given the cross-sectional nature of this study, we cannot establish the causal association between air pollutants and blood pressure, thus further well-designed longitudinal studies are warranted to confirm our results. Second, we collected the demographic information and life style from face-to-face interview by qualified questionnaires, which may subject to recall bias. Third, the air pollutants exposure was based on participants' residential location and did not allow for mobility of them, thus may induce measurement error in exposure assessment. However, studies have indicated that this non-differential exposure misclassification might have biased the effects toward null (68, 69). That is to say if we adopted personal exposure levels, the effect estimates would have been higher than the present results of our study. Fourth, due to the accessible of the resources, we only explored the effect of NDVI, which cannot fully represent participants' residential greenness. Fifth, although we have adjusted most possible confounding factors, we cannot rule out confounding bias from unconsidered factors, such as indoor air pollution, noise, traffic factor and so on. Finally, “white coat effect” may occur during the measurement of blood pressure and thus affect our results.

## Conclusion

Long-term PM_2.5_, PM_10_, and SO_2_ exposure was associated with elevated levels of DBP and MAP. These associations were modified by greenness indices NDVI. Our study may be useful to policy makers to reduce the burden caused by high blood pressure. Meanwhile, our study indicated the importance to increase green space to protect human from adverse air pollutants effects. Participants aged ≥50 years were more susceptible to the hypertensive effect of PM_2.5_ and PM_10_ compared to younger adults. Nevertheless, given the study limitation, further well-designed longitudinal studies are warranted to better assess causal relationships.

## Data availability statement

The datasets presented in this article are not readily available because this dataset is now confidential, but will further available by request to the corresponding author. Requests to access the datasets should be directed to xuqun@ibms.cams.cn.

## Ethics statement

The studies involving human participants were reviewed and approved by the Institutional Review Board of the Institute of Basic Medical Sciences, Chinese Academy of Medical Sciences. The patients/participants provided their written informed consent to participate in this study.

## Author contributions

YM: conceptualization, methodology, software, investigation, validation, writing—original draft, and writing—review and editing. JZ, QZ, YL, and KL: investigation, resources, and validation. MZ and JX: investigation and validation. QX: funding acquisition, writing—review and editing, and supervision. All authors contributed to the article and approved the submitted version.

## Funding

This study was supported by the China Prospective cohort study of Air pollution and health effects in Typical areas (C-PAT) (Grant No. MEE-EH-20190802), the Fundamental Research Funds for the Central Universities (Grant No. 3332019147), Peking Union Medical College Graduate Innovation Fund (No. 2019-1004-02), the China Medical Board (Grant No. 15-230), and the Chinese Academy of Medical Science Innovation Fund for Medical Sciences (Grant No. 2017-I2M-1-009).

## Conflict of interest

The authors declare that the research was conducted in the absence of any commercial or financial relationships that could be construed as a potential conflict of interest.

## Publisher's note

All claims expressed in this article are solely those of the authors and do not necessarily represent those of their affiliated organizations, or those of the publisher, the editors and the reviewers. Any product that may be evaluated in this article, or claim that may be made by its manufacturer, is not guaranteed or endorsed by the publisher.
