# Supplementary material for: Residential greenness attenuated association of long-term air pollution exposure with elevated blood pressure: Findings from polluted areas in Northern China
Source: Front Public Health. 2022 Sep 29;10:1019965. doi: 10.3389/fpubh.2022.1019965 (PMC9557125; doi:10.3389/fpubh.2022.1019965)
Supplement: Supplementary file 1 [file Table_1.docx]

**Supplementary file**

**Residential greenness attenuated association of long-term air pollution exposure with elevated blood pressure: Findings from polluted areas in northern China**

Yayuan Mei^a,b^, Jiaxin Zhao^a,b^, Quan Zhou^a,b^, Meiduo Zhao^a,b^, Jing Xu^a,b^, Yanbing Li^a,b^, Kai Li^a,b^, Qun Xu^a,b,*^

^a^Department of Epidemiology and Biostatistics, Institute of Basic Medical Sciences Chinese Academy of Medical Sciences, School of Basic Medicine Peking Union Medical College, Beijing 100005, China;

^b^Center of Environmental and Health Sciences, Chinese Academy of Medical Sciences, Peking Union Medical College, Beijing 100005, China;

* Correspondence to:

Qun Xu, Ph.D

Department of Epidemiology and Biostatistics, Institute of Basic Medical Sciences Chinese Academy of Medical Sciences, School of Basic Medicine Peking Union Medical College, Beijing 100005, China

Tel.: +86 10 69156403

Fax: +86 10 69156403

E-mail: xuqun@ibms.cams.cn

**Table of contents**

**Tables**

**Table S1.** Results of the association between air pollutants and blood pressure indicators from the three progressive adjustment model.

**Table S2.** Results of the association between NDVI and blood pressure indicators from the three progressive adjustment model.

**Table S3.** Stratified analysis: association between 10-μg/m^3^ increase in ***PM_1_*** and blood pressure indicators by potential modifiers.

**Table S4.** Stratified analysis: association between 10-μg/m^3^ increase in ***PM_10_*** and blood pressure indicators by potential modifiers.

**Table S5.** Stratified analysis: association between 10-μg/m^3^ increase in ***NO_2_*** and blood pressure indicators by potential modifiers.

**Table S6.** Stratified analysis: association between 10-μg/m^3^ increase in ***O_3_*** and blood pressure indicators by potential modifiers.

**Table S7.** Stratified analysis: association between 0.1 increase in ***NDVI*** and blood pressure indicators by potential modifiers.

**Table S8.** The *P* values for the interaction between air pollutants and dichotomous terms for high or low NDVI exposure.

**Figures**

**Figure S1.** Spearman correlations between exposure variables including air pollutants and NDVI.

**Figure S2.** Association between NDVI and blood pressure indicators.

**Figure S3.** Sensitive analysis: association between air pollutants and blood pressure *using the 2-year as the long-term exposure metrics.*

**Figure S4.** Sensitive analysis: association between air pollutants and blood pressure after *excluding outliers.*

**Figure S5.** Sensitive analysis: association between air pollutants and blood pressure after *restricting participants without diabetes, hypertension or dyslipidemia.*

**Figure S6.** Sensitive analysis: association between air pollutants and blood pressure *using the 5-year as the long-term exposure metrics*.

**Table S1. Results of the association between air pollutants and blood pressure indicators from the three progressive adjustment model.**

| Outcome | Model | PM_1_ | PM_2.5_ | PM_10_ | NO_2_ | SO_2_ | O_3_ |
| --- | --- | --- | --- | --- | --- | --- | --- |
| SBP | Model 1 | 0.90 (-1.12, 2.95) | 0.99 (-0.23, 2.21) | 0.73 (-0.14, 1.61) | 0.26 (-2.43, 3.03) | 1.67 (-0.83, 4.24) | -1.47 (-4.5, 1.65) |
|  | Model 2 | 0.77 (-1.18, 2.75) | 0.88 (-0.28, 2.05) | 0.69 (-0.16, 1.54) | 0.18 (-2.43, 2.85) | 1.55 (-0.90, 4.06) | -1.14 (-4.12, 1.93) |
|  | Model 3 | 0.73 (-0.68, 2.16) | 0.77 (-0.08, 1.63) | 0.49 (-0.10, 1.09) | 0.41 (-1.63, 2.50) | 1.08 (-0.70, 2.9) | 0.65 (-1.63, 2.99) |
| DBP | Model 1 | 2.12 (-0.28, 4.57) | **2.80 (1.13, 4.49)** | **1.74 (0.74, 2.75)** | 0.32 (-2.57, 3.31) | **3.51 (1.04, 6.03)** | -2.78 (-5.64, 0.17) |
|  | Model 2 | 1.93 (-0.41, 4.33) | **2.69 (1.05, 4.36)** | **1.70 (0.71, 2.70)** | 0.13 (-2.60, 2.93) | **3.39 (0.98, 5.85)** | -2.57 (-5.35, 0.29) |
|  | Model 3 | 2.09 (-0.08, 4.30) | **2.36 (0.97, 3.76)** | **1.51 (0.70, 2.34)** | 1.44 (-1.69, 4.67) | **3.54 (1.55, 5.56)** | -0.93 (-3.65, 1.85) |
| MAP | Model 1 | 1.43 (-0.60, 3.51) | **1.84 (0.46, 3.24)** | **1.27 (0.35, 2.20)** | 0.16 (-2.41, 2.79) | **2.76 (0.44, 5.13)** | -1.94 (-4.71, 0.92) |
|  | Model 2 | 1.23 (-0.71, 3.21) | **1.66 (0.34, 3.01)** | **1.20 (0.31, 2.10)** | 0.04 (-2.39, 2.52) | **2.61 (0.35, 4.92)** | -1.62 (-4.33, 1.16) |
|  | Model 3 | 1.58 (-0.07, 3.26) | **1.84 (0.74, 2.96)** | **1.17 (0.52, 1.83)** | 0.55 (-1.64, 2.80) | **2.43 (0.71, 4.18)** | -0.41 (-2.64, 1.88) |
| PP | Model 1 | 0.05 (-3.63, 3.87) | 0.36 (-1.71, 2.48) | 0.05 (-1.46, 1.59) | 1.11 (-3.93, 6.42) | -0.83 (-5.29, 3.83) | -1.86(-7.47, 4.09) |
|  | Model 2 | 0.01 (-3.65, 3.80) | 0.36 (-1.69, 2.45) | 0.10 (-1.40, 1.62) | 0.96 (-4.04, 6.23) | -0.84 (-5.29, 3.81) | -1.43 (-7.04, 4.53) |
|  | Model 3 | -0.09 (-3.62, 3.57) | 0.18 (-1.85, 2.25) | -0.27 (-1.74, 1.21) | 1.31 (-3.55, 6.42) | -1.97 (-6.06, 2.31) | 0.73 (-4.56, 6.32) |

Abbreviations: PM_1_, particulate matter with a diameter of <1 µm; PM_2.5_, fine particulate matter of <2.5 µm; PM_10_, particulate matter with a diameter of <10 µm; NO_2_, nitrogen dioxide; SO_2_, sulphur dioxide; O_3_, ozone; SBP, systolic blood pressure; DBP, diastolic blood pressure; MAP, mean arterial pressure; PP, pulse pressure.

**Table S2. Results of the association between NDVI and blood pressure indicators from the three progressive adjustment model.**

|  | Model 1 | Model 2 | Model 3 |
| --- | --- | --- | --- |
| SBP | -0.12 (-0.70, 0.47) | -0.18 (-0.75, 0.40) | -0.08 (-0.49, 0.32) |
| DBP | 0.27 (-0.31, 0.85) | 0.23 (-0.34, 0.80) | 0.10 (-0.41, 0.62) |
| MAP | 0.08 (-0.47, 0.63) | 0.03 (-0.51, 0.57) | 0.11 (-0.32, 0.53) |
| PP | -0.57 (-1.60, 0.48) | -0.63 (-1.66, 0.41) | -0.56 (-1.51, 0.41) |

Abbreviation: SBP, systolic blood pressure; DBP, diastolic blood pressure; MAP, mean arterial pressure; PP, pulse pressure. NDVI, Normalized Difference Vegetation Index.

**Table S3.** **Stratified analysis: association between 10-μg/m^3^ increase in *PM_1_* and blood pressure indicators by potential modifiers.**

| Stratification factors | Percent changes (95% CI) | | | |
| --- | --- | --- | --- | --- |
|  | SBP | DBP | MAP | PP |
| Age |  |  |  |  |
| <50 | 0.81 (-0.23, 1.86) | 0.72 (-0.42, 1.87) | 0.88 (-0.10, 1.87) | 2.08 (-0.21, 4.42) |
| ≥50 | 1.05 (-0.78, 2.92) | **2.94 (0.39, 5.56)** | **2.20 (0.19, 4.25)** | -0.92 (-4.88, 3.21) |
| Sex |  |  |  |  |
| male | 0.19 (-1.41, 1.83) | 1.25 (-0.90, 3.45) | 0.93 (-0.79, 2.67) | -0.79 (-5.21, 3.83) |
| female | 0.25 (-1.36, 1.89) | 1.45 (-0.97, 3.92) | 1.20 (-0.71, 3.14) | -0.01 (-3.76, 3.88) |
| Smoking |  |  |  |  |
| No | 0.59 (-1.02, 2.23) | 1.94 (-0.34, 4.27) | 1.67 (-0.16, 3.55) | 0.16 (-3.18, 3.60) |
| Yes | 0.48 (-1.00, 1.98) | 0.96 (-1.68, 3.67) | 0.70 (-1.01, 2.45) | -0.40 (-4.75, 4.15) |
| Drinking |  |  |  |  |
| No | 0.51 (-1.02, 2.06) | 1.72 (-0.67, 4.18) | 1.33 (-0.48, 3.17) | 0.75 (-2.76, 4.38) |
| Yes | 0.43 (-1.45, 2.34) | 0.60 (-1.28, 2.52) | 0.72 (-1.12, 2.60) | -0.91 (-5.84, 4.28) |

Abbreviations: PM_1_, fine particulate matter of <1 µm; SBP, systolic blood pressure; DBP, diastolic blood pressure; MAP, mean arterial pressure; PP, pulse pressure. The asterisk indicate there is significant difference between the subgroups; no asterisk indicate there is no significant difference between the subgroups.

**Table S4.** **Stratified analysis: association between 10-μg/m^3^ increase in *PM_10_* and blood pressure indicators by potential modifiers.**

| Stratification factors | Percent changes (95% CI) | | | |
| --- | --- | --- | --- | --- |
|  | SBP | DBP | MAP | PP |
| Age |  |  |  |  |
| <50 | 0.39 (-0.03, 0.81) | 0.44 (-0.03, 0.90) | **0.45 (0.05, 0.86)** | 0.70 (-0.15, 1.55) |
| ≥50 | 0.74 (-0.03, 1.51) | **2.11 (1.11, 3.11)*** | **1.67 (0.84, 2.50)*** | -0.50 (-2.17, 1.19) |
| Sex |  |  |  |  |
| male | 0.39 (-0.29, 1.07) | **1.07 (0.17, 1.98)** | **0.70 (0.03, 1.38)** | -0.37 (-2.06, 1.36) |
| female | 0.34 (-0.37, 1.05) | **1.20 (0.28, 2.14)** | **0.93 (0.18, 1.69)** | 0.16 (-1.35, 1.69) |
| Smoking |  |  |  |  |
| No | 0.40 (-0.28, 1.08) | **1.44 (0.57, 2.33)** | **1.08 (0.38, 1.79)** | -0.04 (-1.41, 1.34) |
| Yes | 0.38 (-0.22, 0.99) | 0.84 (-0.18, 1.87) | 0.53 (-0.14, 1.21) | -0.11 (-1.87, 1.68) |
| Drinking |  |  |  |  |
| No | 0.51 (-0.16, 1.19) | **1.16 (0.24, 2.08)** | **0.94 (0.24, 1.65)** | 0.31 (-1.11, 1.75) |
| Yes | 0.31 (-0.44, 1.07) | 0.86 (-0.00, 1.72) | 0.62 (-0.10, 1.33) | -0.46 (-2.35, 1.47) |

Abbreviations: PM_10_, fine particulate matter of <10 µm; SBP, systolic blood pressure; DBP, diastolic blood pressure; MAP, mean arterial pressure; PP, pulse pressure. The asterisk indicate there is significant difference between the subgroups; no asterisk indicate there is no significant difference between the subgroups.

**Table S5.** **Stratified analysis: association between 10-μg/m^3^ increase in *NO_2_* and blood pressure indicators by potential modifiers.**

| Stratification factors | Percent changes (95% CI) | | | |
| --- | --- | --- | --- | --- |
|  | SBP | DBP | MAP | PP |
| Age |  |  |  |  |
| <50 | 0.95 (-0.36, 2.28) | 0.85 (-0.58, 2.29) | 1.00 (-0.22, 2.24) | 2.51 (-0.33, 5.44) |
| ≥50 | 0.55 (-1.81, 2.96) | 2.47 (-1.31, 6.4) | 1.42 (-1.39, 4.31) | 0.79 (-4.79, 6.70) |
| Sex |  |  |  |  |
| male | 0.23 (-2.02, 2.54) | 0.31 (-2.26, 2.96) | 0.40 (-1.71, 2.55) | 1.02 (-4.96, 7.38) |
| female | 0.03 (-2.28, 2.39) | 0.15 (-2.84, 3.23) | 0.35 (-2.09, 2.84) | 1.74 (-3.13, 6.85) |
| Smoking |  |  |  |  |
| No | -0.02 (-2.31, 2.33) | 1.00 (-1.96, 4.04) | 0.59 (-1.72, 2.96) | 0.97 (-3.43, 5.58) |
| Yes | 0.97 (-1.02, 2.99) | -0.06 (-3.71, 3.73) | 0.53 (-1.72, 2.82) | 2.41 (-3.04, 8.17) |
| Drinking |  |  |  |  |
| No | 0.18 (-1.97, 2.38) | -0.11 (-3.06, 2.93) | 0.19 (-2.05, 2.49) | 2.52 (-1.70, 6.93) |
| Yes | 1.11 (-1.57, 3.86) | 0.39 (-1.99, 2.83) | 0.77 (-1.61, 3.20) | 2.3 (-4.84, 9.97) |

Abbreviations: NO_2_, nitrogen dioxide; SBP, systolic blood pressure; DBP, diastolic blood pressure; MAP, mean arterial pressure; PP, pulse pressure. The asterisk indicate there is significant difference between the subgroups; no asterisk indicate there is no significant difference between the subgroups.

**Table S6.** **Stratified analysis: association between 10-μg/m^3^ increase in *O_3_* and blood pressure indicators by potential modifiers.**

| Stratification factors | Percent changes (95% CI) | | | |
| --- | --- | --- | --- | --- |
|  | SBP | DBP | MAP | PP |
| Age |  |  |  |  |
| <50 | 1.03 (-1.00, 3.11) | 0.91 (-1.27, 3.13) | 0.95 (-0.89, 2.82) | 3.10 (-1.40, 7.80) |
| ≥50 | 0.40 (-2.19, 3.05) | -2.19 (-5.26, 0.99) | -1.13 (-3.82, 1.65) | 0.85 (-5.20, 7.30) |
| Sex |  |  |  |  |
| male | -1.15 (-3.75, 1.52) | 0.26 (-2.60, 3.21) | -0.48 (-2.97, 2.07) | -4.08 (-10.22, 2.47) |
| female | 1.10 (-1.67, 3.95) | -0.53 (-3.65, 2.69) | -0.27 (-2.82, 2.35) | 3.57 (-2.33, 9.83) |
| Smoking |  |  |  |  |
| No | 0.93 (-1.68, 3.62) | -1.15 (-3.98, 1.76) | -0.99 (-3.28, 1.35) | 2.25 (-3.03, 7.81) |
| Yes | -0.15 (-2.91, 2.69) | 2.13 (-1.88, 6.30) | 0.75 (-2.03, 3.62) | -4.90 (-12.38, 3.21) |
| Drinking |  |  |  |  |
| No | 0.69 (-1.85, 3.29) | -0.30 (-3.39, 2.88) | -0.09 (-2.49, 2.38) | 2.69 (-2.75, 8.43) |
| Yes | -1.50 (-4.51, 1.62) | -0.28 (-3.23, 2.75) | -1.14 (-3.98, 1.78) | -4.29 (-11.07, 3.00) |

Abbreviations: O_3_, ozone; SBP, systolic blood pressure; DBP, diastolic blood pressure; MAP, mean arterial pressure; PP, pulse pressure. The asterisk indicate there is significant difference between the subgroups; no asterisk indicate there is no significant difference between the subgroups.

**Table S7.** **Stratified analysis: association between 0.1 increase in *NDVI* and blood pressure indicators by potential modifiers.**

| Stratification factors | Percent changes (95% CI) | | | |
| --- | --- | --- | --- | --- |
|  | SBP | DBP | MAP | PP |
| Age |  |  |  |  |
| <50 | 0.18 (-0.51, 0.86) | 0.15 (-0.60, 0.91) | 0.26 (-0.38, 0.90) | 0.29 (-1.37, 1.97) |
| ≥50 | -0.05 (-0.50, 0.41) | 0.31 (-0.27, 0.90) | 0.23 (-0.26, 0.72) | -0.73 (-1.78, 0.33) |
| Sex |  |  |  |  |
| male | -0.25 (-0.79, 0.29) | 0.23 (-0.43, 0.89) | 0.02 (-0.54, 0.60) | -1.13 (-2.44, 0.20) |
| female | -0.09 (-0.60, 0.43) | 0.14 (-0.48, 0.77) | 0.19 (-0.32, 0.71) | -0.47 (-1.58, 0.66) |
| Smoking |  |  |  |  |
| No | -0.08 (-0.55, 0.40) | 0.20 (-0.36, 0.77) | 0.26 (-0.20, 0.73) | -0.59 (-1.58, 0.41) |
| Yes | -0.15 (-0.76, 0.46) | 0.08 (-0.76, 0.92) | -0.01 (-0.67, 0.65) | -1.25 (-2.79, 0.32) |
| Drinking |  |  |  |  |
| No | -0.02 (-0.50, 0.47) | 0.15 (-0.47, 0.76) | 0.17 (-0.32, 0.67) | -0.35 (-1.47, 0.79) |
| Yes | -0.20 (-0.81, 0.40) | 0.24 (-0.44, 0.91) | 0.10 (-0.51, 0.71) | -1.32 (-2.70, 0.07) |

Abbreviations: NDVI, Normalized Difference Vegetation Index; SBP, systolic blood pressure; DBP, diastolic blood pressure; MAP, mean arterial pressure; PP, pulse pressure. The asterisk indicate there is significant difference between the subgroups; no asterisk indicate there is no significant difference between the subgroups.

**Table S8.** **The *P* values for the interaction between air pollutants and dichotomous terms for high or low NDVI exposure**

| Interaction term | | *P* values | | | |
| --- | --- | --- | --- | --- | --- |
|  |  | SBP | DBP | MAP | PP |
| PM_1_ | *NDVI | 0.432 | **0.006** | **0.036** | 0.340 |
| PM_2.5_ |  | 0.779 | **0.005** | 0.050 | 0.104 |
| PM_10_ |  | 0.851 | 0.065 | 0.212 | **0.048** |
| NO_2_ |  | 0.597 | **0.046** | 0.241 | **0.043** |
| SO_2_ |  | 0.612 | **0.021** | **0.047** | 0.273 |
| O_3_ |  | 0.629 | 0.273 | 0.361 | 0.792 |

Abbreviations: NDVI, Normalized Difference Vegetation Index; SBP, systolic blood pressure; DBP, diastolic blood pressure; MAP, mean arterial pressure; PP, pulse pressure. The asterisk indicate there is significant difference between the subgroups; no asterisk indicate there is no significant difference between the subgroups.

**
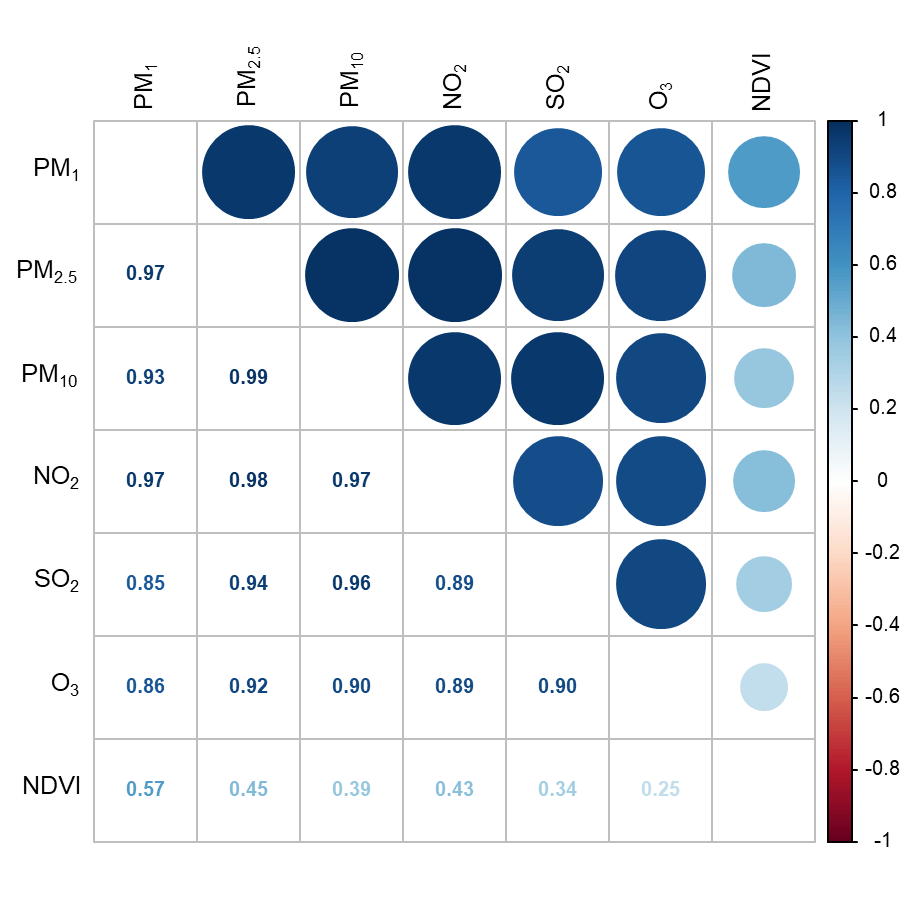
Figure S1. Spearman correlations between exposure variables including air pollutants and NDVI.**

Abbreviations: PM_1_, particulate matter with a diameter of <1 µm; PM_2.5_, fine particulate matter of <2.5 µm; PM_10_, particulate matter with a diameter of <10 µm; NO_2_, nitrogen dioxide; SO_2_, sulphur dioxide; O_3_, ozone; NDVI, Normalized Difference Vegetation Index.


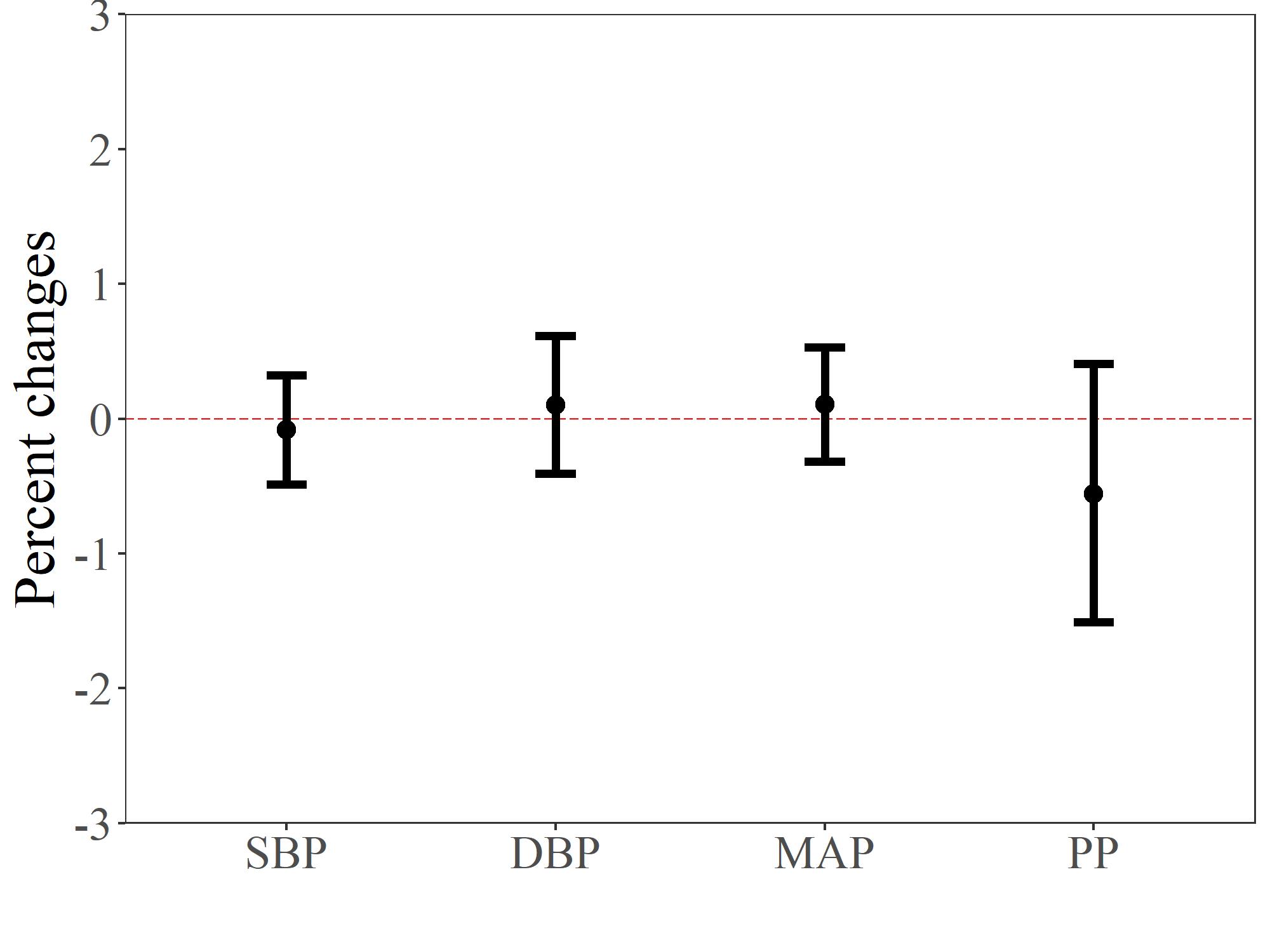
**Figure S2. Association between NDVI and blood pressure indicators.**

Abbreviations: NDVI, Normalized Difference Vegetation Index; SBP, systolic blood pressure; DBP, diastolic blood pressure; MAP, mean arterial pressure; PP, pulse pressure.

**Figure S3. Sensitive analysis: association between air pollutants and blood pressure using the 2-year as the long-term exposure metrics.**


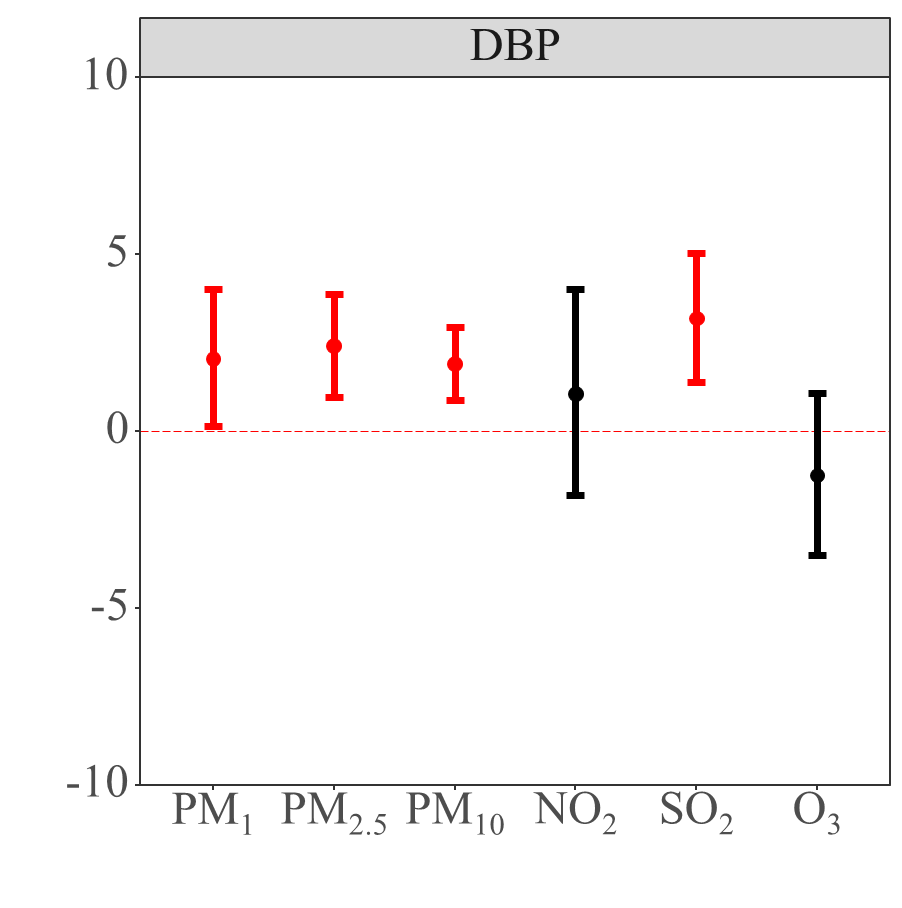

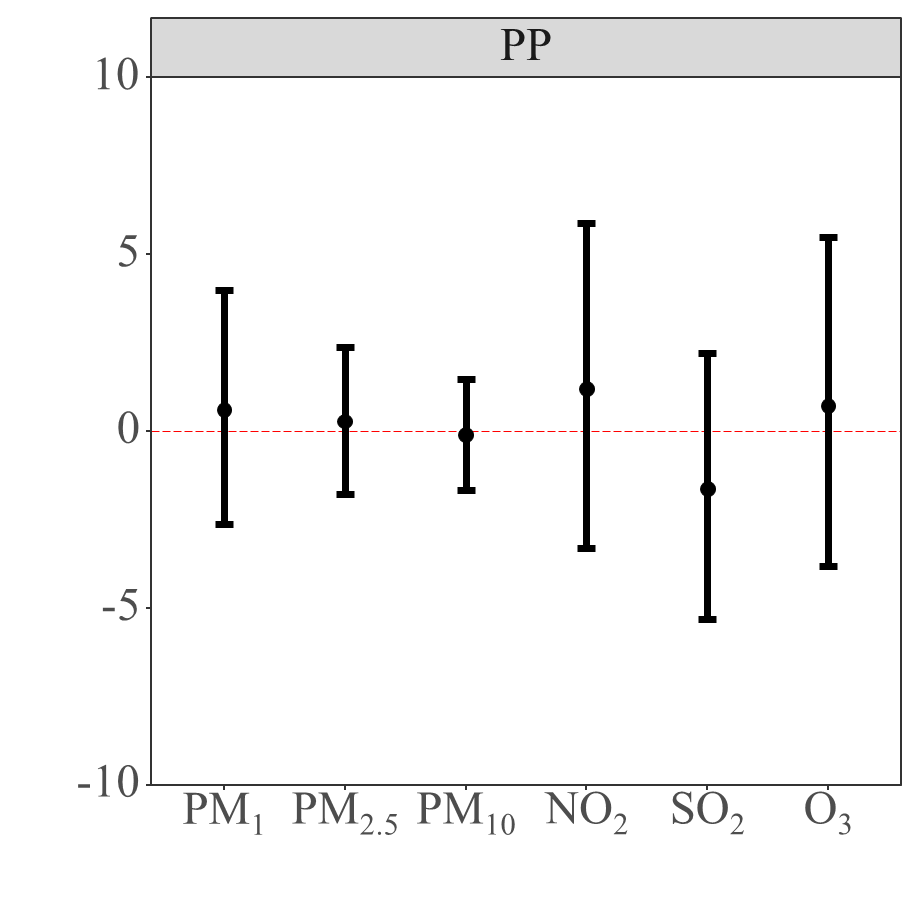

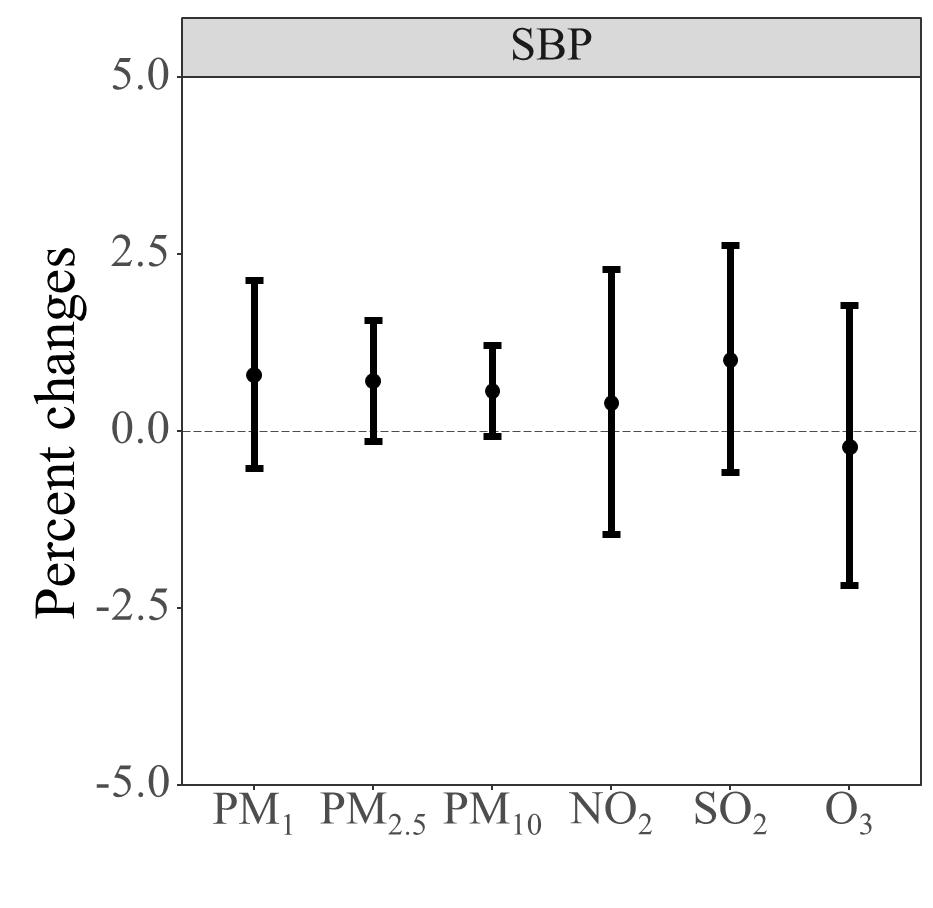

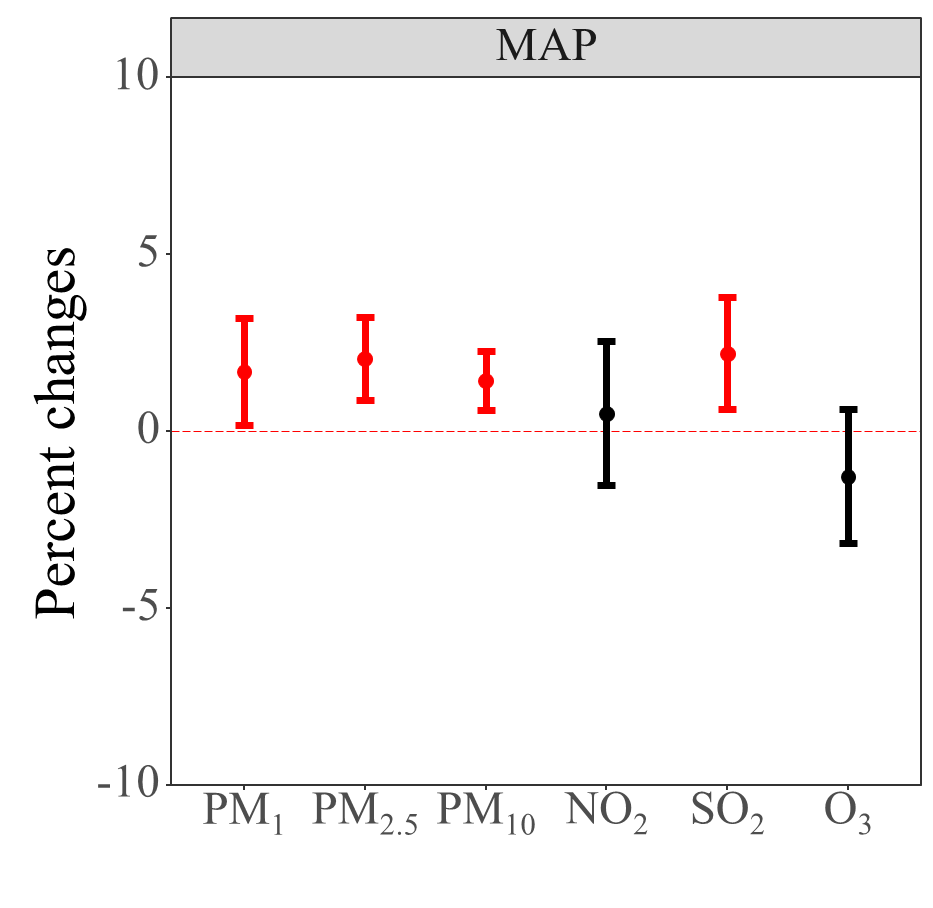


Abbreviations: PM_1_, particulate matter with a diameter of <1 µm; PM_2.5_, fine particulate matter of <2.5 µm; PM_10_, particulate matter with a diameter of <10 µm; NO_2_, nitrogen dioxide; SO_2_, sulphur dioxide; O_3_, ozone; SBP, systolic blood pressure; DBP, diastolic blood pressure; MAP, mean arterial pressure; PP, pulse pressure.

**Figure S4. Sensitive analysis: association between air pollutants and blood pressure after excluding outliers.**


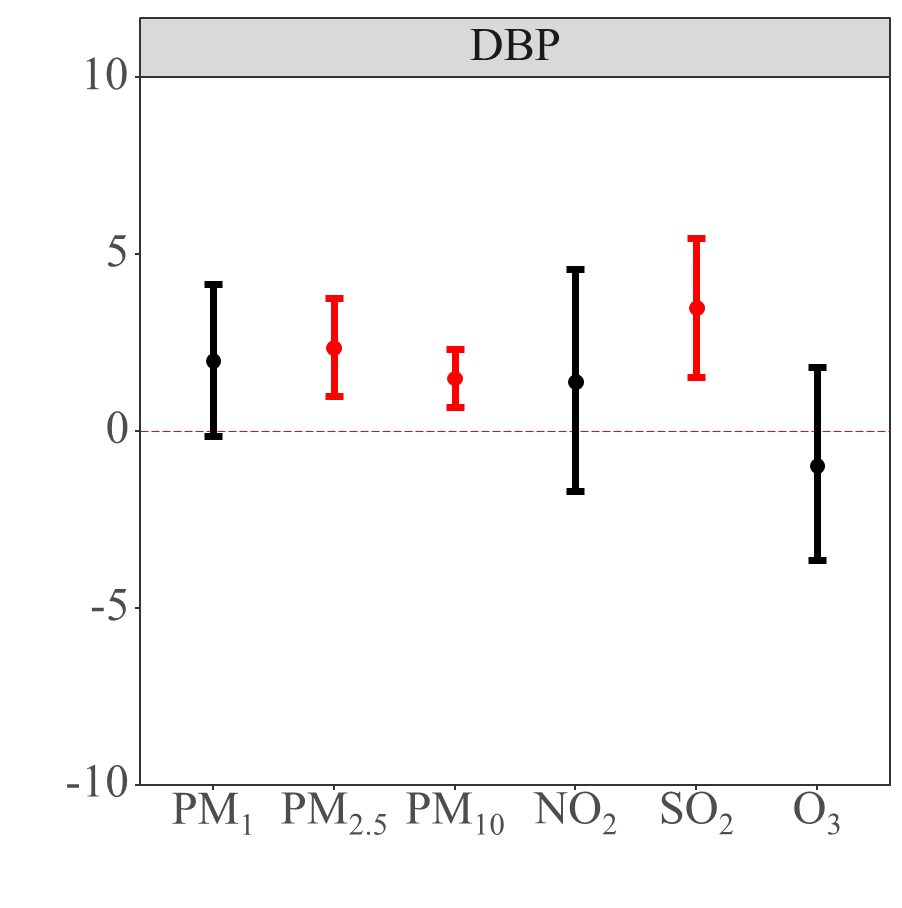

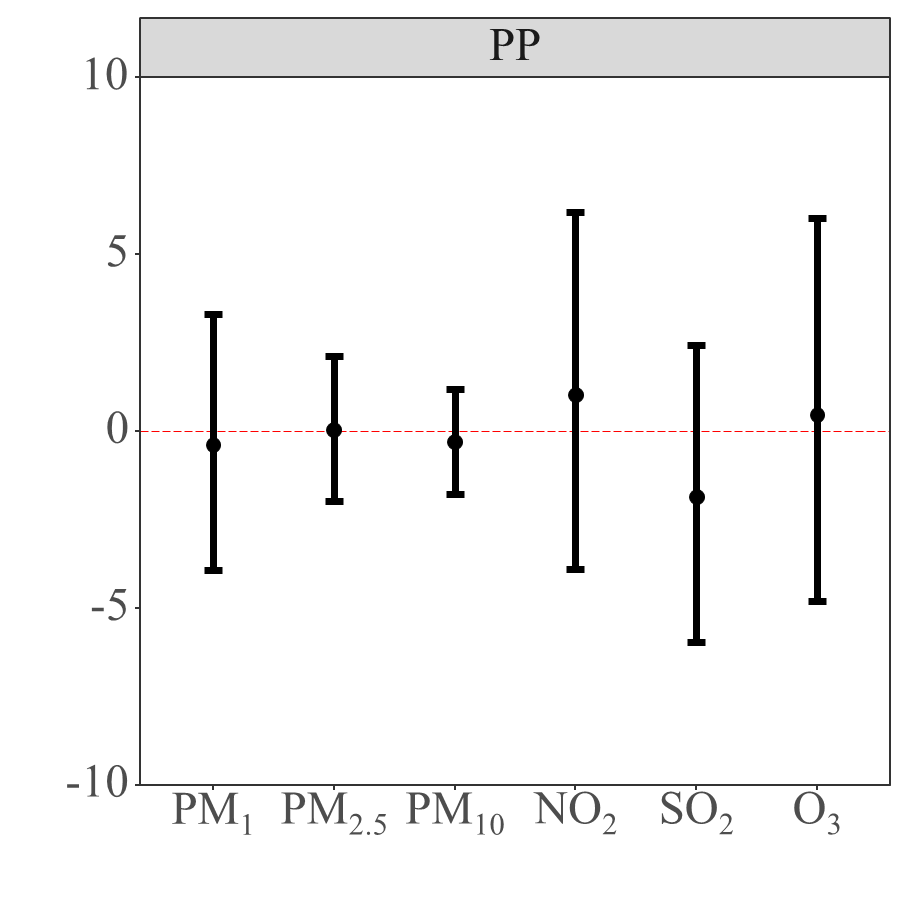

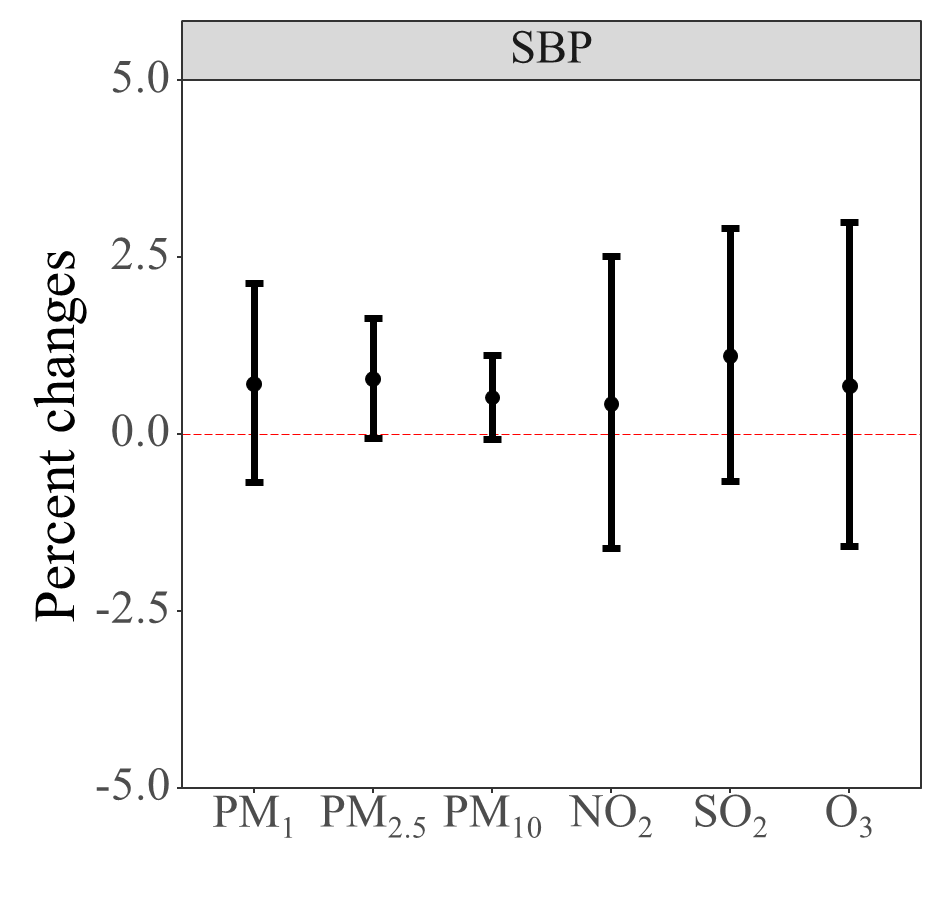

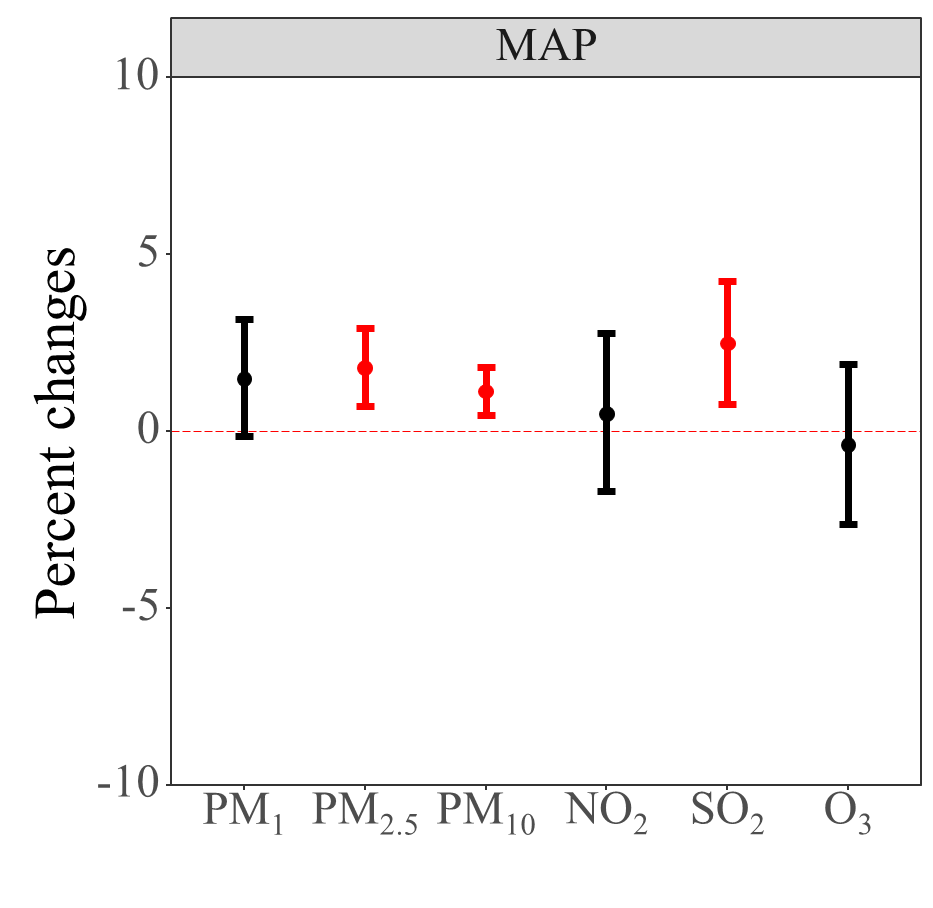


Abbreviations: PM_1_, particulate matter with a diameter of <1 µm; PM_2.5_, fine particulate matter of <2.5 µm; PM_10_, particulate matter with a diameter of <10 µm; NO_2_, nitrogen dioxide; SO_2_, sulphur dioxide; O_3_, ozone; SBP, systolic blood pressure; DBP, diastolic blood pressure; MAP, mean arterial pressure; PP, pulse pressure.

**Figure S5. Sensitive analysis: association between air pollutants and blood pressure after restricting participants without diabetes, hypertension or dyslipidemia.**


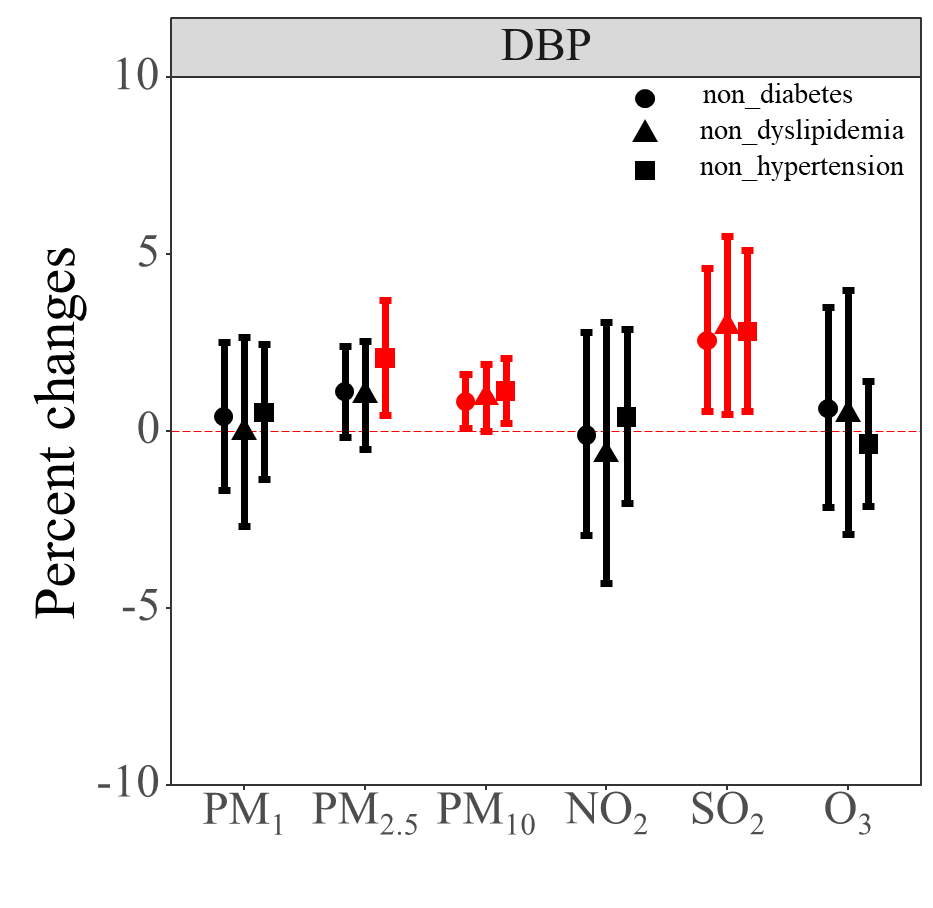

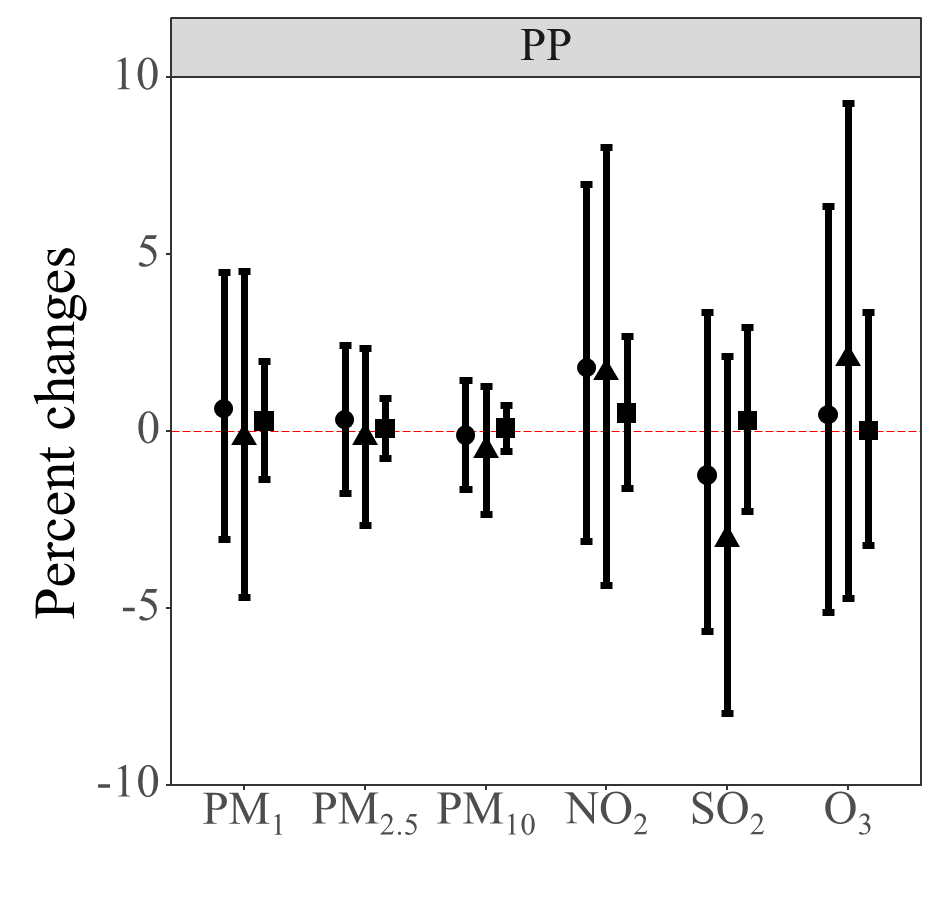

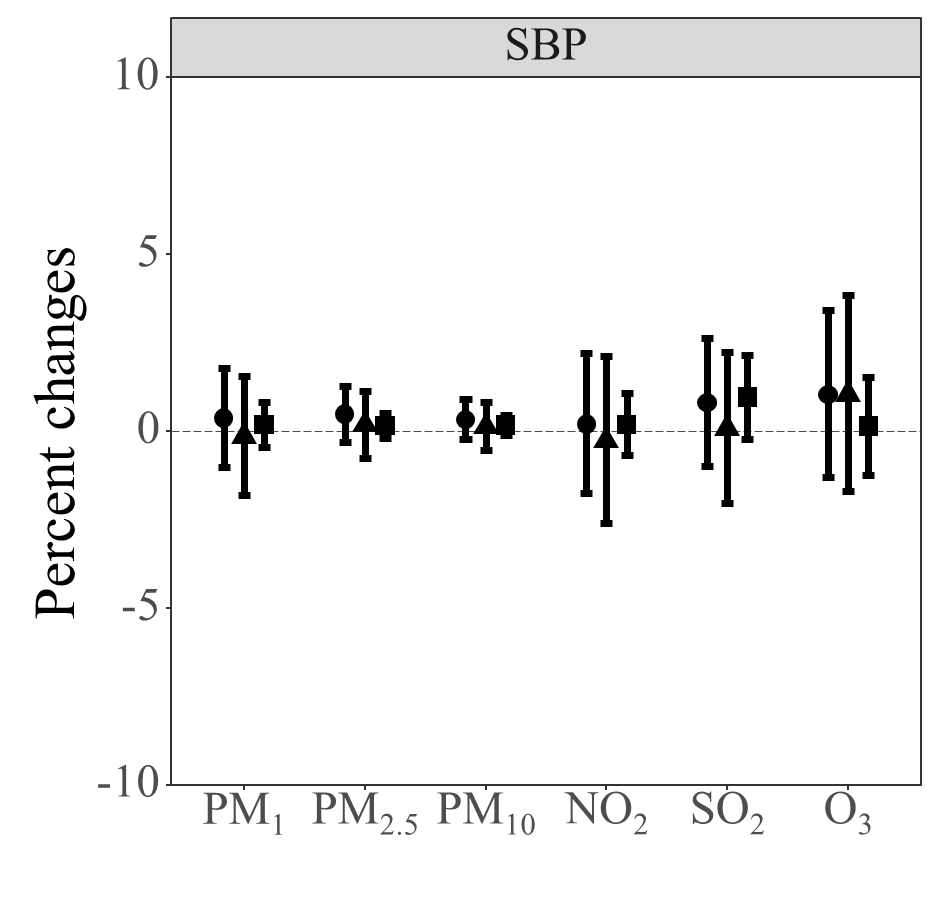

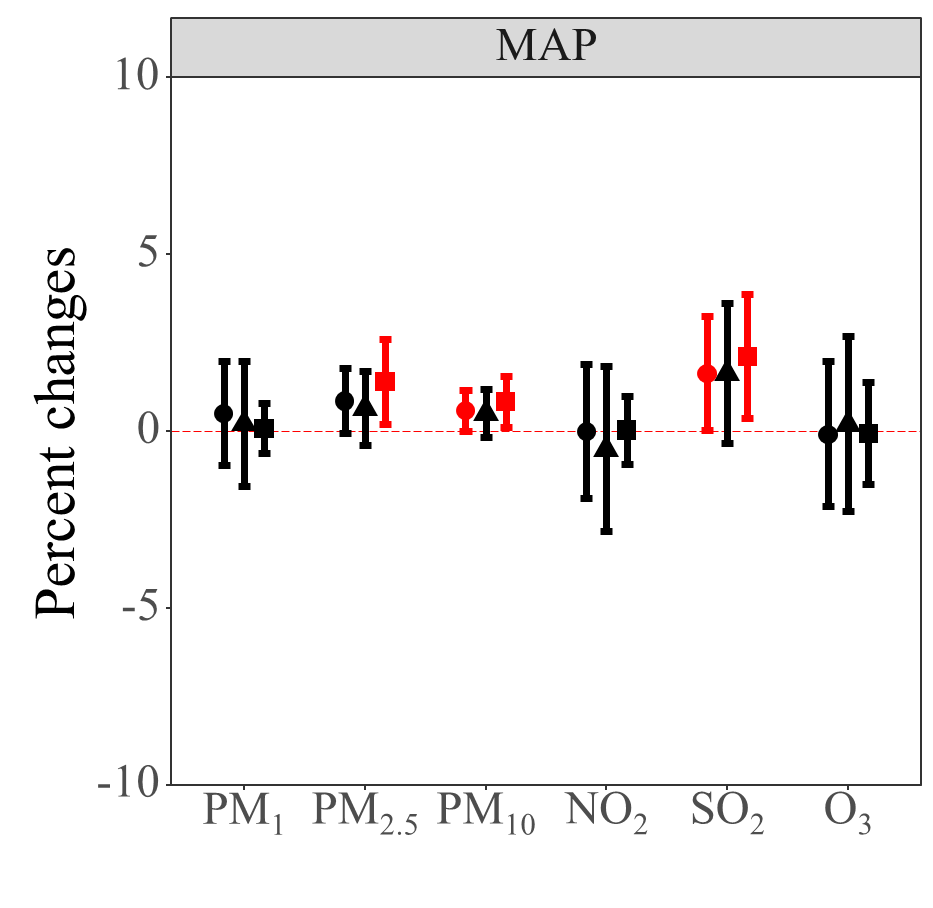


Abbreviations: PM_1_, particulate matter with a diameter of <1 µm; PM_2.5_, fine particulate matter of <2.5 µm; PM_10_, particulate matter with a diameter of <10 µm; NO_2_, nitrogen dioxide; SO_2_, sulphur dioxide; O_3_, ozone; SBP, systolic blood pressure; DBP, diastolic blood pressure; MAP, mean arterial pressure; PP, pulse pressure.

**Figure S6. Sensitive analysis: association between air pollutants and blood pressure using the 5-year as the long-term exposure metrics.**


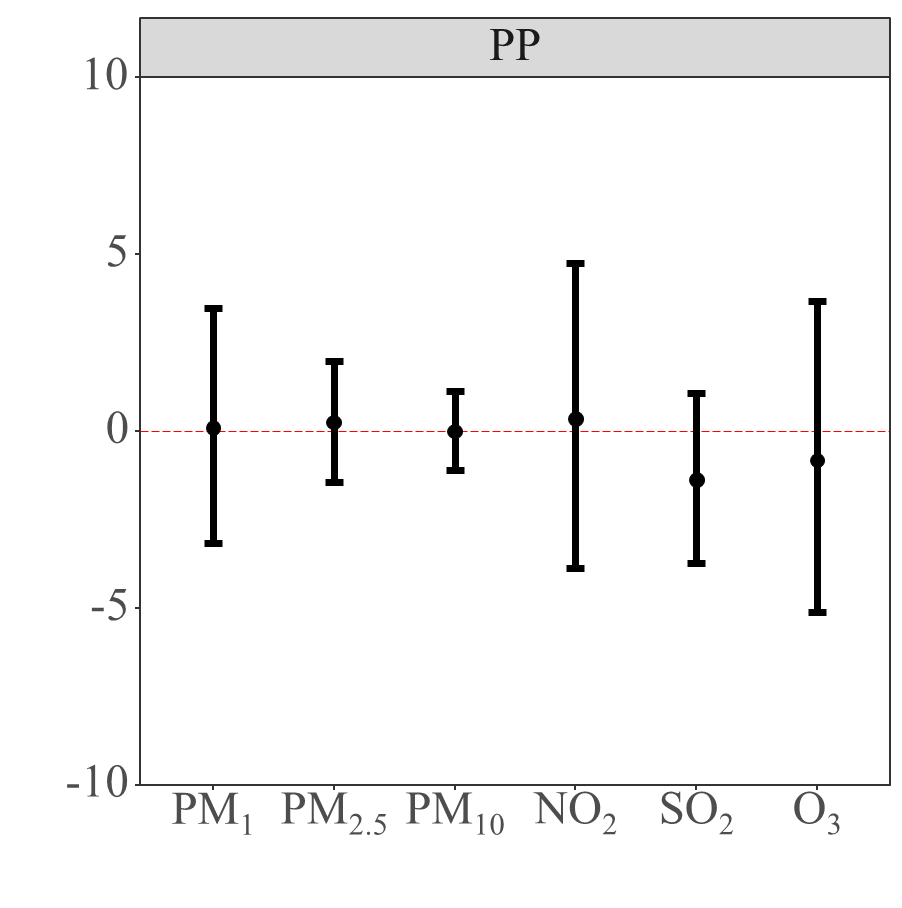

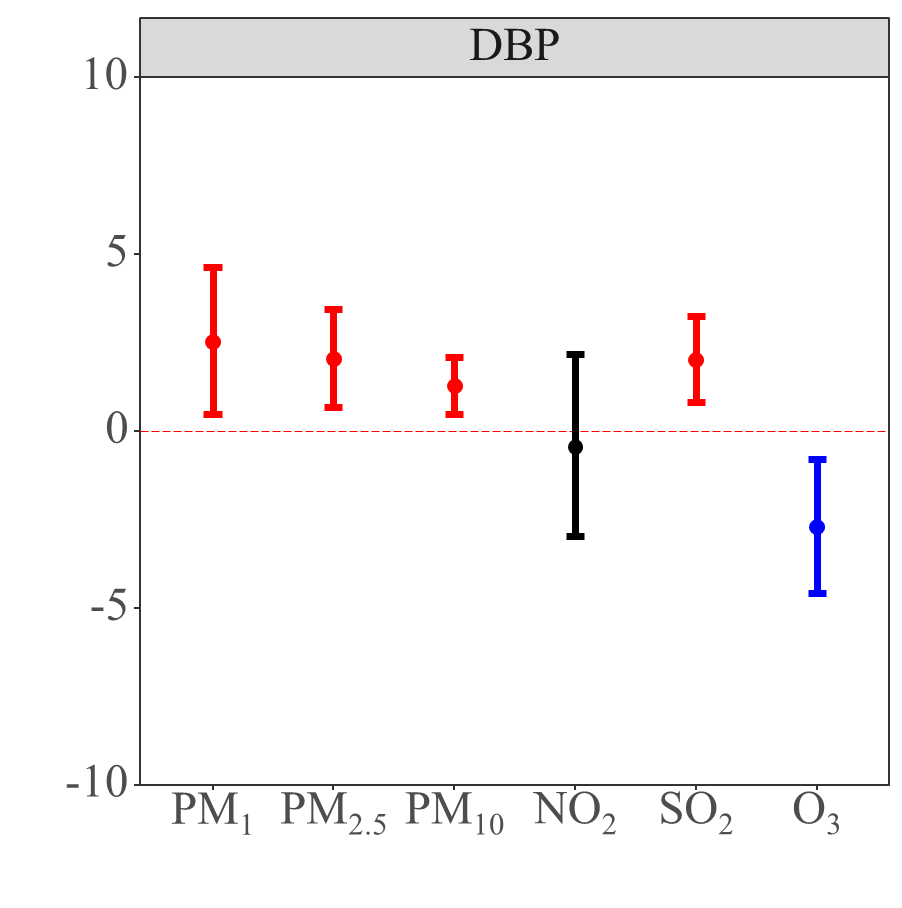

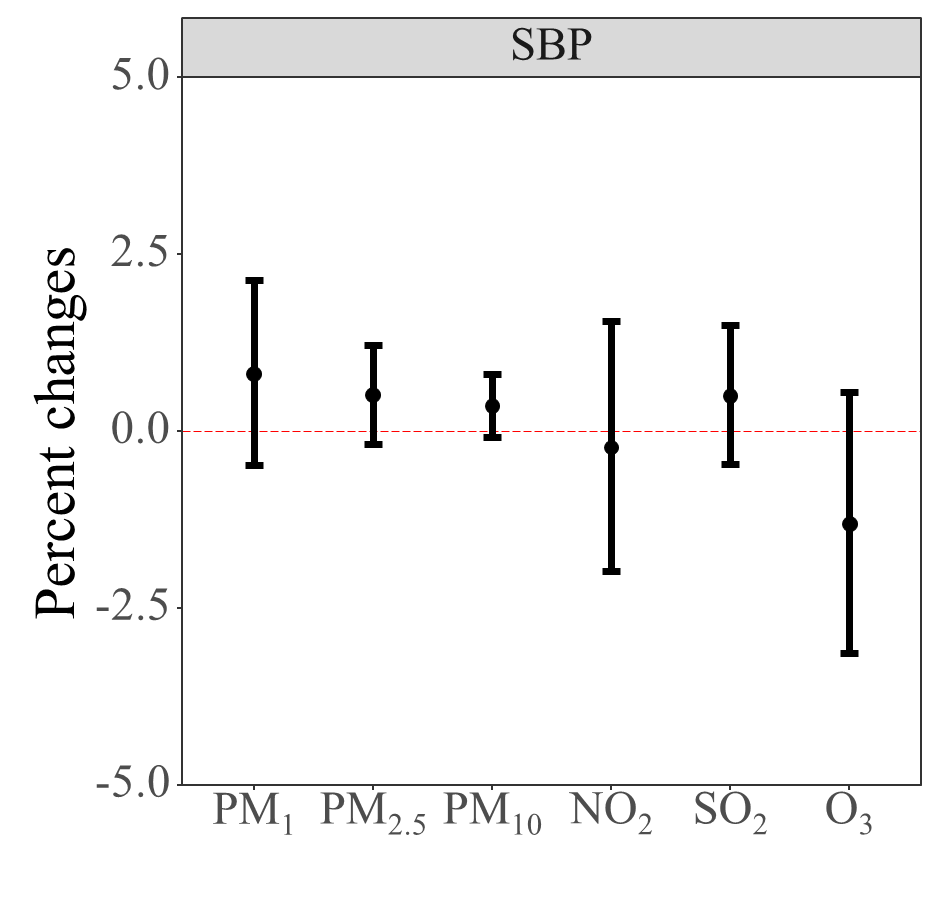

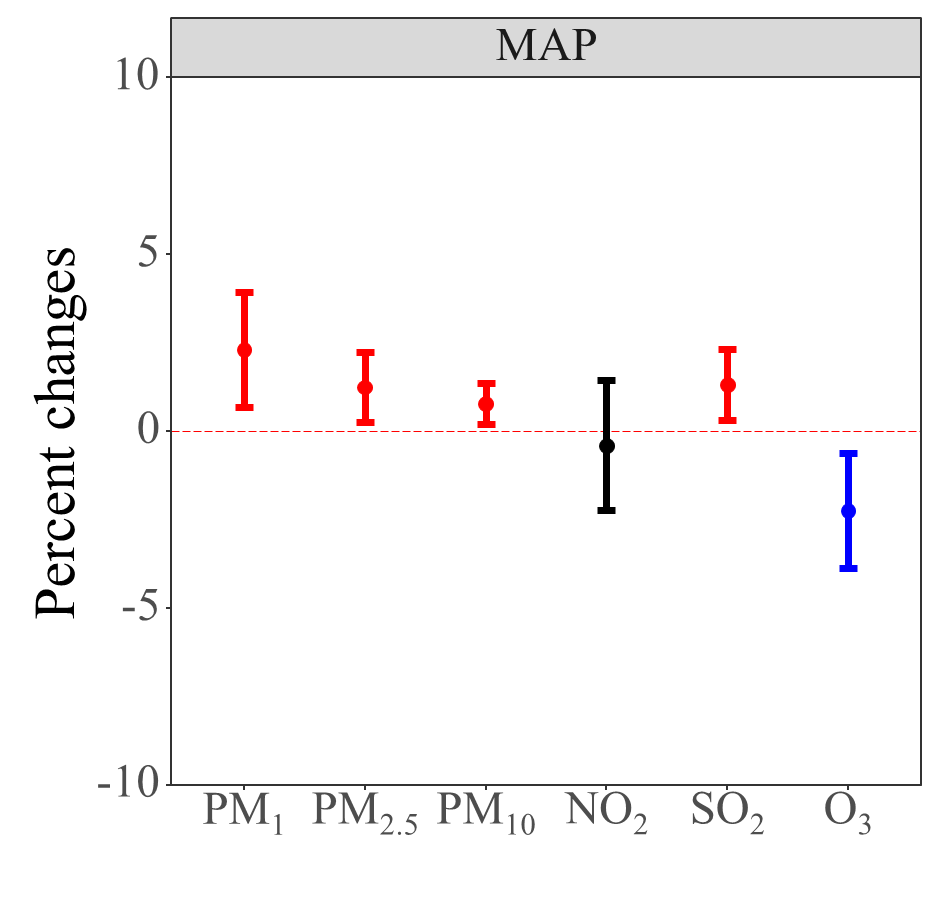


Abbreviations: PM_1_, particulate matter with a diameter of <1 µm; PM_2.5_, fine particulate matter of <2.5 µm; PM_10_, particulate matter with a diameter of <10 µm; NO_2_, nitrogen dioxide; SO_2_, sulphur dioxide; O_3_, ozone; SBP, systolic blood pressure; DBP, diastolic blood pressure; MAP, mean arterial pressure; PP, pulse pressure. Please note that the long-term exposure windows for PM_1_ were 4-year due to the data restriction for PM_1_. The exposure windows for the rest air pollutants were 5-year.
